# Molecular and Clinical Investigation of COVID-19: From Pathogenesis and Immune Responses to Novel Diagnosis and Treatment

**DOI:** 10.3389/fmolb.2022.770775

**Published:** 2022-05-19

**Authors:** Narjes Riahi Kashani, Javid Azadbakht, Hassan Ehteram, Hamed Haddad Kashani, Hassan Rajabi-Moghadam, Ejaz Ahmad, Hossein Nikzad, Elahe Seyed Hosseini

**Affiliations:** ^1^ Anatomical Sciences Research Center, Institute for Basic Sciences, Kashan University of Medical Sciences, Kashan, Iran; ^2^ Gametogenesis Research Center, Kashan University of Medical Sciences, Kashan, Iran; ^3^ Department of Radiology, Faculty of Medicine, Kashan University of Medical Sciences, Kashan, Iran; ^4^ Department of Pathology, School of Medicine, Kashan University of Medical Sciences, Kashan, Iran; ^5^ Department of Cardiovascular Medicine, Kashan University of Medical Sciences, Kashan, Iran; ^6^ Department of Pathology, Michigan Medicine, University of Michigan, Ann Arbor, MI, United States

**Keywords:** coronavirus, immune system, vaccine, antiviral, respiratory syndrome

## Abstract

The coronavirus-related severe acute respiratory syndrome (SARS-CoV) in 2002/2003, the Middle East respiratory syndrome (MERS-CoV) in 2012/2013, and especially the current 2019/2021 severe acute respiratory syndrome-2 (SARS-CoV-2) negatively affected the national health systems worldwide. Different SARS-CoV-2 variants, including Alpha (B.1.1.7), Beta (B.1.351), Gamma (P.1), Delta (B.1.617.2), and recently Omicron (B.1.1.529), have emerged resulting from the high rate of genetic recombination and S1-RBD/S2 mutation/deletion in the spike protein that has an impact on the virus activity. Furthermore, genetic variability in certain genes involved in the immune system might impact the level of SARS-CoV-2 recognition and immune response against the virus among different populations. Understanding the molecular mechanism and function of SARS-CoV-2 variants and their different epidemiological outcomes is a key step for effective COVID-19 treatment strategies, including antiviral drug development and vaccine designs, which can immunize people with genetic variabilities against various strains of SARS-CoV-2. In this review, we center our focus on the recent and up-to-date knowledge on SARS-CoV-2 (Alpha to Omicron) origin and evolution, structure, genetic diversity, route of transmission, pathogenesis, new diagnostic, and treatment strategies, as well as the psychological and economic impact of COVID-19 pandemic on individuals and their lives around the world.

## Highlights

• The emergence of novel types of COVID-19 can be a serious health threat for humans.
• Genetic variability in certain genes involved in the immune system, encoding human leukocyte antigen (HLA) A, B, and C, may affect susceptibility to and severity of SARS-CoV-2 infection.
• One vaccine or treatment option alone cannot immunize people with genetic variabilities against various strains of SARS-CoV-2 in different areas of the world.
• Among different virus vectors, the adeno-associated virus can be used to deliver the CRISPR/Cas13d system to infected lung cells in SARS-CoV-2 patients.

## Introduction

From the outset of the twenty-first century, three zoonotic β-coronaviruses (CoVs) have crossed the inter-species barriers, infected humans, and caused severe fatal pneumonia. Severe acute respiratory syndrome coronavirus (SARS-CoV) first appeared in 2003 (Poutanen et al., 2003; Zhong, 2004), Middle East respiratory syndrome coronavirus (MERS-CoV) emerged in 2012 (Lu L. et al., 2013) and a novel β-coronavirus appeared in Hubei province, China, in December 2019 (Wu and McGoogan, 2020; Zhu et al., 2020). The novel β-coronavirus was named 2019-novel coronavirus (2019-nCoV), and the infection it causes was called COVID-19 disease by the World Health Organization (WHO) on 12 January 2020 (Du Toit, 2020; Gralinski and Menachery, 2020). Afterward, on 11 February 2020, the International Committee on Taxonomy of Viruses (ICTV) study group renamed it severe acute respiratory syndrome coronavirus 2 (SARS-CoV-2) (Gorbalenya et al., 2020). The WHO revised the state of Public Health International Emergency (30 January 2020) to a pandemic (11 March 2020) (Du Toit, 2020). Despite global efforts to control this serious pandemic, it rapidly spread worldwide and continued to rise. Two years after the COVID-19 appearance, the number of confirmed infections reported to WHO has exceeded 435 million, with a death toll of 5,952,215 until March 2022 (World Health Organization, 2022a). COVID-19 can be asymptomatic, mild, or severe or lead to death. Most of the patients infected by COVID-19 clinically manifest with mild symptoms, such as dry cough, breathing difficulty, fever, and nausea (Huang C. et al., 2020). However, others suffer from acute and severe pneumonia, which can develop with serious complications such as septic shock, pulmonary non-cardiogenic edema, acute respiratory distress syndrome (ARDS), organ failure, and damage to the lung parenchyma (Chan W. et al., 2020; Huang C. et al., 2020; Lu H. et al., 2020). The incubation period for COVID-19 is usually <14 days. Advanced age is also associated with increased mortality. Patients having any medical comorbidities (obesity, diabetes, tumor, or heart, lung, and/or kidney diseases) have a greater risk of developing severe COVID-19 and higher mortality rates (World Health Organization, 2022b). Although, no specific animal has been identified to date as an early natural source of novel COVID-19 disease, it is suggested that several animal species or mammalians or birds, including bats, snakes, pangolins, poultry, marmots, and turtles sold at Seafood Wholesale Market in Wuhan’s Huanan, were probably associated with SAR-CoV-2 (Chung et al., 2020; Huang P. et al., 2020; Yang Y. et al., 2020). Recent comparative genomic analyses combined with evolutionary tree synthesis assumed that bat coronaviruses such as Bat-CoV-RaTG13a are closest relatives to SARS-CoV-2 with 96% similarity at the genomic level and phylogenetic homology and transmitted to humans through some unknown potential intermediated hosts. The case fatality rate (CFR) of the novel SARS-CoV-2 (2%–4%) is relatively lower than that of SARS-CoV and MERS-CoV (Li W. et al., 2005a; Cui et al., 2019; Li and Shi, 2019; Zhou P. et al., 2020). However, genetics and clinical evidence suggests structural, genetic, and epidemiological similarity of novel COVID-19 to those of SARS and MERS, making the previous findings applicable to COVID-19. Thus far, various SARS-CoV-2 strains (from Alpha to Omicron) with high transmissibility, infectivity, and mortality rates have emerged secondary to a high rate of recombination, S1/S2/RBD mutation, deletion, and substitutions during convergent evolution (Andreata-Santos et al., 2022). This review aims to provide the reader with the most recent information on SARS-CoV-2 special features, such as diverse strains, the host-pathogen interaction, virus pathogenicity, treatment strategies, the mechanisms by which virus evades immune system, transmission route, genetic variability in human immunity-related genes, and finally, different host immune responses. These data may serve as a reference for more precise and comprehensive studies: firstly, to psychologically minimize the potential negative effects of some misinformation about this not-fully-understood virus on people on a worldwide scale and, secondly, to build up a base knowledge as a first step to design an efficient vaccine for COVID-19 (Cui et al., 2019).

## Origin and Evolution of SARS-CoV-2

A profound knowledge of how an animal coronavirus jumped across inter-species boundaries to infect humans will considerably help prevent the following zoonotic outbreaks. At present, the source and intermediate host of SARS-CoV-2 are unknown, and no compelling evidence currently shows that any domestic animal can transmit SARS-CoV-2 to other animals, including humans. Sequencing 2019-nCoV in different patients showed almost 99.9% sequence identity, suggesting that novel SARS-CoV-2 emanated from one source within a very limited period of time (Zhang T. et al., 2020; Zhou P. et al., 2020). Comparisons of this SARS-CoV-2 with other coronaviruses in origin and evolution might help us find its initial reservoirs. In this regard, investigating the evolutionary relationship of the receptor-binding domain (RBD) sequence in spike protein, the cleavage site of S-protein in SARS-CoV-2, and identification of the early sequence, intermediate host its receptor would be of great help in understanding the origin of the virus. The SARS-phylogenetic analysis demonstrated that novel SARS-Cov-2 is closely related to the bat SARS-like CoVs such as bat-SL-CoVZC45 and bat-SL-CoVZXC21 (Lu R. et al., 2020; Zhou P. et al., 2020). Phylogenetic tree and whole-genome analysis also showed that SARS-CoV-2 shares above 85% nucleotide sequence homology with previous SARS-CoVs, including SARS/MERS CoVs (Dhama et al., 2020; Li W. et al., 2005a). Furthermore, the phylogenetic tree based on S-proteins and the RBD of SARS-CoV-2 showed 74% similarity at the RBD level and 76% S-protein similarity to these SARS-CoVs, respectively (Jaimes et al., 2020; Walls et al., 2020). Because of the high degree of similarity of RBD in spike protein, both novel SARS-CoV-2 and previous SARS-CoVs are likely to use the same receptor for angiotensin-converting enzyme 2 (ACE2) to enter host cells (Jaimes et al., 2020). In addition, the following cell culture studies illustrated that the spike protein of the new SARS-CoV-2 employs the human angiotensin-converting enzyme 2 (ACE2), the same receptor as what SARS-CoV uses for entry (Letko et., 2020). However, the most notable difference between 2019-nCoV S and SARS-CoVS is an insertion in the S1/S2 protease cleavage site that prompts an “RRAR” fur in the recognition site in SARS-CoV-2 compared to the single arginine in SARS-CoV [28,29]. On the contrary, 96% nucleotide homology has been demonstrated between SARS-CoV-2 and Bat-CoV-RaTG13 in a study conducted in Yunnan, China [18, 30]. It has also been shown that SARS-CoV-2 shares 97.43% identity in the spike protein and 89.57% identity in the amino acid sequence of RBD with Bat RaTG13 [18–19,31] as well as the highest similarity in ORF1ab (98.55) and nucleocapsid protein (N) (99.05), respectively (Li Q. et al., 2020). Such a high degree of sequence similarity suggests that the SARS-CoV-2 is more closely related to the Bat-CoVRaTG13 than the other SARS-CoVs (Li C. et al., 2020). These findings also suggest that bats can still be considered a potential source as they host the closest relative of SARS-CoV-2, similar to the case for SARS-CoV and MERS-CoV (Huang J.-M. et al., 2020). Moreover, other animal species might serve as intermediate hosts between bats and humans. SARS-CoV-2 was first reported on 31 December 2019, when most bat species were hibernating in Wuhan. Besides, a variety of other animals but not bats were vended at the Huanan seafood market. Additionally, SARS-CoV-2 has less than 90% sequence identity with its nearest relatives, bat-SL-CoVZC45 and bat-SL-CoVZXC21, which reveals that these Bat-like CoVs are not direct ancestors of 2019-nCoV. What is more, bats have been recognized as natural sources of SARS-CoV and MERS-CoV pathogens, which have been passed on to humans *via* some intermediate hosts such as palms or civets (Y. Guan et al., 2003) or dromedary camels (Azhar et al., 2014), respectively. Therefore, given that the first group of patients infected with COVID-19 were in contact with wild animals sold at a Chinese seafood market, it is suggested that bats are the initial hosts of SARS-CoV-2, which in turn has been transmitted to humans by an unknown wild animal host(s) (Lau et al., 2005; Chan J. F.-W. et al., 2020). Previous studies on the possible intermediate hosts, with regard to viral receptor-binding domains (RBD) and host receptors, have suggested that snakes, pangolins, and turtles may also serve as potential intermediate hosts in transmitting the virus to humans (Liu Z. et al., 2020). With 93.2% nucleotide and 94.1% amino acid identity to SARS-CoV-2, pangolin CoV has been suggested as the most closely related to SARS-CoV-2. Moreover, Pangolin-CoV shows 92.8% nucleotide and 93.5% amino acid identity to Bat RaTG13 (Lam et al., 2020; Zhang T. et al., 2020). However, some Pangolin-CoV genes, including orf1b, the spike (S) protein, orf7a, and orf10, share higher amino acid sequence homology with SARS-CoV-2 than RaTG13 genes. Comparative analysis of SARS-CoV-2, Bat RaTG13, and pangolin CoV in RBD and five essential amino-acid residues engaged with human ACE2 revealed that SARS-CoV-2 has 96.68% RBD identity with pangolin CoV and 89% RBD similarity with Bat RaTG13 (Zhang T. et al., 2020). Furthermore, Pangolin CoV has only 85% RBD similarity with the Bat RaTG13. These findings indicate that Pangolin-CoV is highly similar to SARS-CoV-2 compared to RaTG13. Interestingly, these five key amino-acid residues have a major role in human-to-human and cross-species transmission. However, only one amino acid is different between Pangolin-CoV and SARS-CoV-2, which does not belong to the five cardinal residues engaged in the interaction with human ACE2. Contrarily, RaTG13 accommodates 17 amino acid residues different from SARS-CoV-2, of which four belong to the key amino acid residues (Zhang T. et al., 2020). These findings also provide more evidence to support the hypothesis that chances are higher for pangolin CoV to endure the host defenses and infect humans than Bat RaTG13 (Zhang T. et al., 2020). Besides, the nucleocapsid protein (N-protein) is the most plenteous and conserved protein in coronaviruses, including SARS-CoV-2, Pangolin-CoV, and RaTG13. Phylogenetic analysis showed that the nucleocapsid protein (N-protein) of SARS-CoV-2 and RaTG13 contains four dissimilar amino acids (37S/P, 215G/S, 243G/S, and 267A/Q), while their S-proteins differ by as many as 33 amino acids. It has been shown that the SARS-CoV-2 virus has a very distinctive peptide (PRRA) insertion located at position 680 of the S-proteins, which may be associated with the cellular proteases and proteolytic cleavage, and affects the host’s transmissibility. Bat RaTG13 does not have this insertion in its S-protein (Wong S. K. et al., 2004; Li X. et al., 2020c; Ji W. et al., 2020). These findings further support the hypothesis that Pangolin-CoV is a highly possible intermediate host involved in cross-species spread and transmission to humans compared with bat RaTG13 or other SARS-CoVs. For cross-species spread and transmission to humans, SARS-CoV-2 must acquire a cleavage site or undergo some mutations, insertions, and deletions occurring at its spike protein, near the S1–S2 junction, allowing for optimal and improved binding to human-like ACE2 (Ji W. et al., 2020). The interaction of five key amino acid residues of S-protein with the angiotensin-converting enzyme-2 (ACE2) receptor is thought to be critical for human-to-human and cross-species transmission of SARS-CoV-2. It is also possible for the SARS-CoV-2 to jump into humans through an animal host with an ACE2-encoding gene similar to the human orthologous (allowing natural selection to proceed efficiently) (Andersen et al., 2020). Similarity plot analysis of bat, pangolin, and SARS-CoV-2 nucleotide sequence also indicated possible recombination in S-protein of SARS-CoV-2. Both Pangolin-CoV and RaTG13 do not have the fur in the recognition sequence motif at the S1/S2 cleavage site of the S-protein as observed in SARS-CoV-2 (Andersen et al., 2020). These findings suggest that SARS-CoV-2 is a recombinant evolved virus of Bat-CoV and Pangolin-CoV with some genetic mutations and recombination in the spike protein gene as a result of natural selection. In fact, some homologous recombination has happened between bat and pangolin CoVs, triggering cross-species transmission of SARS-CoV-2, leading to the evolution that increases its adaptability during the outbreak (Huang J.-M. et al., 2020). This indicates that SARS-CoV-2 might gain optimized ACE2 proteins from an intermediate host such as a bat to facilitate its entry into host cells and suggests that the SARS-CoV-2 S-protein RBD–ACE2 host receptor interaction mediates infection in humans and other animals (Zhou P. et al., 2020). In SARS-CoV-2, some mutations have also been detected in five genes of S, N, ORF8, ORF3a, and ORF1ab, of which about 42% are non-synonymous mutations (Tang et al., 2020). Because of the global spread of SARS-CoV-2, its amino acid sequence has also changed significantly, resulting in increased viral diversity in some SARS-CoV-2 infected patients. This explains the probable cross-species transmission, adaptation of viruses to the human body, human-to-human transmission, and the viral genome evolution in the human population (Shen et al., 2020). Structural studies and biochemical experiments have demonstrated a high affinity for SARS-CoV-2 RBD toward human ACE2 and other species with high receptor homology. Notably, the high-affinity binding of the SARS-CoV-2 spike protein to human ACE2 is most likely the result of natural selection on a human-like ACE2. There is a strong body of evidence in the literature that SARS-CoV-2 might not be a purposefully manipulated laboratory-based virus (Wan et al., 2020).

### Coronavirus: Genome Structure and Classification

Coronaviruses are enveloped, non-segmented positive-sense RNA viruses containing a very large RNA (∼26–32 kb) surrounded by a symmetrical nucleocapsid (Song et al., 2019). They are the largest group of viruses belonging to the Riboviria realm, the idovirales order, including the Coronaviridae, Arteriviridae, and Roniviridae families. The Arteriviridae family are phylogenetically classified into four genera: the Alpha, Beta, Gamma, and Delta coronaviruses (α, β, γ, and δ) (Chandra and Awasthi, 2020). Each genus is further subdivided into linage subgroups of A, B, C, and D. Human CoVs consist of HCoV-229E and NL63, MERS-CoV, SARS-CoV, HCoV-OC43, and HCoV-HKU1 (Chandra and Awasthi, 2020). Four α-coronaviruses of HCoV-229E, HCoV-NL63, HCoV-OC43, and HCoV-HKU1 are associated with mild symptoms in humans, while two human β-coronaviruses of SARS-CoV (Peiris et al., 2003) and MERS-CoV cause severe disease (Xu et al., 2004). The newly emerged SARS-CoV-2 is the seventh human coronavirus, belonging to β-coronaviruses and lineage B subgenus. β-Coronaviruses (including SARS-CoV-2) are classified by the club-like spikes (S-proteins) that project from their outer surface, their large RNA genome, and their replication strategy. Human SARS-CoV-2 genome consisted of 29,903 nucleotide-based RNA molecules (Papanikolaou et al., 2022) with at least ten open reading frames (ORFs), encoding 27 proteins (including 15 nonstructural proteins, 4 structural proteins, and 8 auxiliary proteins). The first ORF (ORF1a/b) forms about two-thirds of viral RNA and encodes two nonstructural polyproteins involved in the formation of viral replicas transcriptase complex. Other ORFs on the remaining one-third of the genome encode four main structural proteins of spike surface glycoprotein (S), envelope (E), nucleocapsid (N), and membrane or matrix (M) proteins. They also encode for sixteen non-structural proteins (NSP1–NSP16) virus’ critical molecules such as helicase and RNA-directed RNA polymerase, which participate in viral replication and translation and facilitate virus entry into the cells (Papanikolaou et al., 2022). The most important feature of coronaviruses is the heavily glycosylated spike glycoprotein (S) (∼150 kDa), located on the surface of CoVs to help them enter target cells. Spike mediates viral entry into host cells through homodimers protruding from the viral surface (Tortorici and Veesler, 2019; Walls et al., 2020). Spike S encompasses two subunits of S1 (N-terminal) and S2 (C-terminal), which create a unique crown-like formation (corona) on virion’s surface. S1 subunit acts as the main receptor-binding domain (RBD) and recognizes and binds to the host cell surface receptor, whereas the S2 domain is involved in the fusion mechanism between cell membrane and virus (Li F. et al., 2005; Ge et al., 2013; Lu G. et al., 2013; Raj et al., 2013; Gui et al., 2017; Yuan et al., 2017; Letko et al., 2020; Walls et al., 2020). These two subunits (S1 and S2) have a major role in viral infection and pathogenesis and are critical targets for antiviral neutralizing antibodies. SARS-CoV-2 uses angiotensin-converting enzyme 2 (ACE2) as a receptor to enter target cells (Hoffmann et al., 2020a; Yao H.-P. et al., 2020). ACE2 is expressed on human nasal epithelial cells, lung, spermatogonia, Sertoli, gastric, duodenal, and rectal epithelial cells (Song et al., 2018). ACE2 is a substrate for membranous attachment, activating the S1 and S2 subunits. Then, ACE2–SARS-CoV-2 cell complex triggers intracellular signaling transduction affecting hypoxia regulatory molecules (Tsiambas et al., 2020). Specific proteases, such as furin, trypsin, cathepsin, or serino-protease (transmembrane serine protease 2-TMPRSS2), are involved in the virus entry into the cell, leading to the intracellular infection signal (Coutard et al., 2020; Lukassen et al., 2020). As a host cell protease, furin cleaves the S-protein into two separate polypeptides: the S1 and S2 subunits. The S1 subunit contains the receptor-binding domain (RBD) (with 193 amino acid residues), which contains two subdomains of the core and external portions (Wong S. K. et al., 2004). The RBD core subdomain is responsible for the formation of S trimer particles (Bosch et al., 2003), and the external subdomain with two exposed loops on the surface binds to the ACE2 receptor (Raj et al., 2013). Six RBD amino acids of SARS-CoV-2, including L455, F486, Q493, S494, N501, and Y505, are essential for binding to ACE2 receptors and determining the host range of SARS-CoV-like viruses. Spike RBD has all the structural information needed for virus attachment to the host receptor ACE2 (Andersen et al., 2020). The receptor-binding domain (RBD) in the spike protein is the most variable part of the coronavirus genome (Andersen et al., 2020), which might be influenced by positive selection. Five of these six residues differ between SARS-CoV-2 and SARS-CoV (Raj et al., 2013).

### Different Variants of Novel COVID-19

Like other viruses, SARS-CoV-2 evolves over time. SARS-COV-2 has undergone many mutations since its earlier detection in Wuhan at the end of 2019. Different SARS-COV-2 variants such as Alpha (α), Beta (β), Gamma (γ), Delta (δ), and recently Omicron variant (B.1.1.529) have appeared by a broad spectrum of recombinations, point mutations, deletions, and amino acid substitutions, particularly in spike RBD, and RBM of novel SARS-CoV-2. Beta, Gamma, and Delta variants have been associated with disease severity and higher virulence but lower pathogenicity. WHO classified SARS-CoV-2 variants into three main categories: variants of concern (VOCs), variants of interest (VOIs), and variants under monitoring (VUMs) (Andreata-Santos et al., 2022). The four previously reported VOCs are Alpha (B.1.1.7), Beta (B.1.351), Gamma (P.1), and Delta (B.1.617.2) variants, whereas the recent variant, Omicron (B.1.1.529), was firstly recognized in South Africa on 26 November 2021, designated as the fifth VOC (He X et al., 2021). It seems that the SARS-CoV-2 virus tries to survive through some variants with//ORF deletions with a low level of activity. Furthermore, under the pressure of natural selection and evolution, point deletions sometimes lessen the viral spread (Akkiz, 2021). However, some of the mutations enhance the ability of the virus to spread and transmit across species and infect multiple cell types leading to a variety of human diseases. Typically, various point mutations (substitutions) and specific deletions have occurred in the S1/S2 domains and RBD areas of RNA genomic sequences in SARS-CoV-2, resulting in the emergence of a new set of SARS-CoV-2 variants. Major (non)synonymous mutations affecting the RBD region in novel SARS-CoV-2 include N501Y, E484K, L452R, and K417N/T (Papanikolaou et al., 2022; Rahimi et al., 2021; Winger and Caspari, 2021). P681H/R substitution has been recognized in S1/S2 furin cleavage site, D614G and G142D in S1 and V1176F, A701V, and T20N on the S2 region. Interestingly, critical deletions (ΔН69/ΔV70/Δ156/Δ157/ΔΥ144/ΔL242/ΔА243/ΔL244) influencing the non-RBD S1 region increase the transmission ability and infectivity of the virus and negatively affect the potency of immunity that neutralizing antibodies provide (decreased serum neutralization titles) (Papanikolaou et al., 2022). However, frequent deletions observed in other regions, including open reading frames (ORF) 7 and 8, surprisingly lead to a low replication load and strong response to mAbs. Various mutations of D614G, N501Y, E484K-Q, K417N/T, and L452R are associated with a significantly strong hACE2 binding affinity, elevated viral load production, increased human-to-human transmissibility and infectivity, enhanced disease severity, and immune escape against vaccine and antibody therapeutic strategies due to low immune response rates (Daniloski et al., 2021; Gobeil et al., 2021; Nelson et al., 2021). All of these mutations were predominantly found in Alpha, Beta, Gamma, Delta, Theta, and Omicron variants. Amino acid D614G substitution (glycine for aspartic acid) in spike occurred in all of the five VOCs. D614G mutation demonstrates a higher load of infectious virus in the upper respiratory tract and an increased replication and transmissibility in SARS-COV-2 variants. The combination of deletions of D614G and E484Q only in the Delta variant granted the virus a significantly increased infectivity and transmissibility and empowered the virus as the response rate to mAbs and targeted vaccines reduced substantially. The Omicron SARS-CoV-2 variant also shares N501Y with the Alpha, Beta, and Gamma variants. This mutation is believed to enhance the binding affinity between spike and angiotensin-converting enzyme 2 (ACE2) and enhance transmissibility (Papanikolaou et al., 2022; Kumar et al., 2021). Recent evidence indicates that the Omicron variant is probably more infectious than the Delta and Beta variants (Pulliam et al., 2021; Lippi et al., 2022)). It has been reported that the Omicron variant has at least 35 mutations in its spike (S) protein compared with early SARS-CoV-2. Generally, 15 of the 29 substitutions located in the receptor-binding domain (RBD) are the early target for neutralizing the monoclonal antibody. Moreover, 10 of those on the receptor-biding motif (RBM), involved in recognizing the human angiotensin-converting enzyme 2 (ACE2) receptors, are suggested to increase the spread and virulence of the Omicron variant. These findings suggest that the monoclonal antibodies approved by the Food and Drug Administration (FDA) may be less effective against the Omicron variant. Although the impact of Omicron on morbidity and mortality is still unknown, the number and combination of mutations/deletions/insertions in this recent variant is impressively associated with high transmissibility, infectivity, and possibly increased re-infection rates (Andreata Santos et al., 2022).

### L and S Types of SARS-CoV-2 (SNP)

Population-based genetic analyses of SARS-CoV-2 genomes indicated that this virus has evolved into two major types, L and S, by means of two different SNPs that show nearly complete linkage across the viral strains’ sequence. Although the L type (∼70%) is more prevalent than the S type (∼30%), the S type was the ancestral strain. Although the L type was more prevalent in the early stages of the SARS-CoV-2 outbreak in Wuhan, the frequency of the L type decreased after early January 2020. It is likely that human intervention may have exerted more severe selective pressure on the L type, leading it to be more aggressive, and spread more quickly. On the contrary, the frequency of the S type, which is evolutionarily older and less aggressive, might have relatively increased due to the lower selective pressure imposed on it. A combination of these findings strongly supports an urgent need for further immediate and comprehensive studies on genomic data, epidemiological data, and clinical symptoms of COVID-19 patients, facilitating the development of effective drugs and vaccines against the virus and aiding us in predicting when and where potential epidemics may occur and preventing their tolls (Huang J.-M. et al., 2020; Wang W. et al., 2020).

## Basic Virology and Pathogenesis of SARS-CoV-2

Coronavirus encompasses a large family of viruses that may cause severe respiratory disease. The early site of SARS-CoV-2 infection and its pathogenesis is still unknown. For betacoronaviruses such as SARS-CoV-2, cell entry is the mainstay of cross-species transmission. SARS-CoV-2 binds to ACE2 receptors on alveolar epithelial cells, infects the lower respiratory tract, and causes lethal and severe pneumonia in humans (Fan et al., 2020). The process of SARS-CoV-2 infection consists of virus attachment to the cell surface, receptor involvement, protease cleavage, and membrane fusion. Cell entry depends on the binding of the viral spike S glycoprotein (subunit S1) to the host receptor ACE2 in SARS-CoV-2 [61] and DPP4 in MERS-CoV (Millet and Whittaker, 2014; Ou et al., 2016). In fact, the S-protein-receptor interaction is the first determinant for coronavirus to infect host species. After binding to the host receptor, the virus must access the host cell cytosol. This is generally done through a two-step process of acid-dependent cleavage of S-protein by the host serine protease of TMPRSS2 or other proteases. This cleavage will activate S-protein by its conformational changes, leading to the fusion of the virus to the cellular membranes. The cleavage of the SARS-CoV-2 S-protein occurs at two sites: 1) at the junction site of S1 and S2, a.k.a. S-protein priming, which is important for separating the RBD and the fusion domains of the S-protein; 2) at the S2 site, immediately upstream of the fusion peptide in the S2 subunit, releasing the fusion peptide and causing the virus-membrane fusion (Fehr and Perlman, 2015; Walls et al., 2020). These processes produce two subunits, including an N-terminal S1 that recognizes the cell surface receptor and a C-terminal S1 that promotes the fusion of the viral envelope with the cellular membrane. Notably, the cleavage site sequence can determine the zoonotic potential of coronaviruses. The S-protein interacts with the host receptor ACE2, mediating the receptor-binding domain (RBD) region of the S-protein through a conformational rearrangement (Millet and Whittaker, 2014; Ou et al., 2016). The site of RBD within coronavirus S-protein varies according to the type of the virus. Some viruses (e.g., MHV) have the RBD at the N-terminus of their S1 region, while in others (e.g., SARS-CoV), the RBD is located at the C-terminus of their S1. The fusion of the virus S-protein and host receptor ACE2, which occurs within acidified endosomes (plasma membrane in MHV), is of pivotal importance for the entry of the virus into cells and for inducing the humoral immune response during infection (Fehr and Perlman, 2015; Kirchdoerfer et al., 2016; Yang X. et al., 2020). Cleavage at S2 exposes a fusion peptide, which is inserted into the membrane. Then, the joining of two heptad repeats in S2 forms an antiparallel six-helix bundle (Li F. et al., 2005), which causes the fusion of virus and cellular membranes, resulting in the viral genome release into the cytoplasm. Lack of an adequate and timely immune response against the infection or immunosuppression in infected patients enables rapid viral replication and spread, which initiates critical and deadly stages of the disease, and severe pneumonia, lung damage, and subsequent respiratory failure will ensue (Zheng, 2020). The high binding affinity between ACE2 and the S-protein in SARS-CoV-2 (∼15 nM and 10–20-fold higher than SARS-CoV S) is thought to play a significant role in the human-to-human transmission of SARS-CoV-2 and predicts disease severity in humans (Kuba et al., 2005; Li W. et al., 2005b). Additionally, high expression and activation of serine protease TMPRSS2 at S1/S2 subunits probably is an important determinant of the virus’s tendency to enter the infected cells, viral pathogenesis, transmissibility, and spread among humans (Tortorici et al., 2019). Notably, the ACE2 expression protects against lung injury and is downregulated by SARS-CoV-2; it would thus be interesting to find out whether SARS-CoV-2 interferes with the ACE2 expression (Li, 2008). Moreover, the capability of the virus to engage with ACE2 from different animal species appears to reflect host susceptibility to SARS-CoV infection and explains zoonotic spillover and numerous SARS-CoV-2 human-to-human transmission events reported to date (Muus et al., 2020).

### Sex–Age-Based Differences in Susceptibility to SARS-CoV-2 Infection Sex Difference

A higher mortality rate among older males (≥65 years) suggests a gender and age-dependent difference in susceptibility to SARS-CoV-2 infection and disease outcomes. Epidemiological data from previous COVs and the recent COVID-19 pandemic revealed that male patients demonstrate a more severe disease and increased mortality than females on a global scale (Karlberg et al., 2004). Furthermore, immunological differences suggest that females have a rapid and aggressive innate and adaptive immune response to combat the invading virus, while the reduced antiviral response in males may lead to more susceptibility to severe diseases. The enhanced antiviral response in females results in a reduced viral RNA copy number and reduced expression of viral antigens in a sex hormone-dependent manner. Thus, it is assumed that sex differences in COVID-19 are represented in the early viral infections and hormonal signaling pathways. In SARS-CoV-2, more infection severity in men is associated with increased plasma cytokine levels of the innate immune system, such as IL-8 and IL-18. In contrast, less infection severity in females corresponds with higher T-cell activation. Furthermore, increases in TNF-α and IL-6 immunological activation are significant and independent predictors of severity and mortality in COVID-19 (Viveiros et al., 2021). Typically, genetic, epigenetic, and hormonal factors and the immune system activity are major factors explaining the gender and age differences in SARS-CoV-2 infection. Two major factors in sex-specific immune responses to COVID-19 infection are different expression levels of immune-related genes such as ACE2 located on the X chromosome, and the sex-specific steroid hormones, androgen, and estrogen, which regulate different immune responses in men and women (Markle and Fish, 2014; Asselta et al., 2020). The SARS-CoV-2 virus uses angiotensin-converting enzyme 2 (ACE2) and transmembrane protease serine 2 (TMPRSS2) to facilitate infection. A meta-analysis study indicated that increased expression levels of ACE2, TMPRSS2, and CTSL in specific cell types are correlated with advanced age and male gender. It has been suggested that the cell-type-specific expression or co-expression of ACE2 and proteases, as the mediators of SARS-CoV-2 viral entry, may affect susceptibility, severity, and transmissibility of COVID-19, as well as certain aspects of the epidemiology, and clinical course of the disease (Channappanavar et al., 2017; Muus et al., 2021)*.* Further investigations are needed to understand whether gender, age, and comorbidity are risk factors for COVID-19 infection and death (Liu et al., 2010; Wei et al., 2020).

### X Chromosome

A recent paper highlighted an association between specific ACE1 I/D genotypes and differences in clinical characteristics of COVID-19 patients. This can be due to the hACE2 gene located on chromosome X (band Xp22.2), which is a crucial molecule involved in the immune response. Aggressive clinic-pathological phenotypes of male patients can be explained by chromosome X-linked genes modifications (Tsiambas et al., 2020). It is suggested that the different expression levels of the genes on the X chromosome may affect the susceptibility to and the severity of SARS-CoV-2 infection. Certain genes on the X chromosome regulate immune responses by encoding proteins such as human leukocyte antigens (HLA), ACE2 receptor, Toll-like receptors (TLR7, TLR8), cytokine receptors (IL2RG and IL13RA2), and a transcription factor for regulatory T cells (FOXP3) (Libert et al., 2010; Case et al., 2012; Conti and Younes, 2020). The Y chromosome also possesses various gene regulatory properties and polymorphisms that influence sex-based susceptibility to viral infection (Yang et al., 2010). As a host cell receptor for SARS-CoV-2, ACE2 is one of the major sex-based genes on the X chromosome. The expression level of ACE2 is strongly upregulated by estrogen and androgen and repressed by inflammatory cytokines and T2D (which increases with age and chronic diseases). There is a strong association between ACE2 expression and COVID-19 infection, susceptibility, severity, and fatality. Higher expression of ACE2 in males compared to females is one of the factors related to the severe symptoms and even death of COVID-19 infection (Liu et al., 2010; Fischer et al., 2015). In females, the X-inactivation mechanism (XCI) is the main factor for the sex-dependent expression of ACE2. Females have two X chromosomes, whereas males carry one X and one Y chromosome. Many immune-associated genes are X-linked, and females have two copies of these genes. Females benefit from a large reserve of proteins provided by the two X chromosomes. The X-inactivation mechanism in females balances the double allelic dosage of genes on the X chromosome by the epigenetic silencing of one of the X chromosomes in the early development process. The ACE2 gene is an X-linked gene that resides in the Xp22.2 region of the X chromosome and escapes X chromosome inactivation, which may confer a “double-dosage” of ACE2 mRNA. This mechanism leads to cellular mosaicism in women. ACE2 expression is dependent on sex hormones. In addition, certain mutations in X chromosome genes such as ACE2 in COVID-19 patients have been detected in some lung cells in females, whereas all the cells in males will exhibit the risky allelic variant (Liu et al., 2010; Bianchi et al., 2012; Case et al., 2012; Markle and Fish, 2014). Like the ACE2 receptor, the protease TMPRSS2 is crucial for SARS-CoV-2 entry into the host cells (Zhou P. et al., 2020). Both genes mediate COVID-19 infection through their sex-dependent expression in the lung cell. Higher expression of TMPRSS2 in male lung cells promotes SARS-CoV-2 entry to cells *via* membrane fusion; additionally, the TMPRSS2 expression is induced by androgen/estrogen stimulation (Souyris et al., 2018). Furthermore, some genetic variants in the 3’ region of the gene TMPRSS2 identified in lung cells might have a significant impact on the TMPRSS2 expression and its catalytic activity, leading to more severe disease (Jin et al., 2020). The Toll-like receptor 7 (TLR7) gene, encoded on the X chromosome, is another gene that may escape X inactivation, resulting in a higher expression level of TLR7 in female immune cells, which causes more cytokine production against viral infection. Stronger Th1 immune responses in females also cause a lower susceptibility to the COVID-19 infectious pathogen than in males (Pinheiro et al., 2011; Markle and Fish, 2014). However, sex hormones may modulate ACE2 expression.

### MicroRNAs and Long Non-Coding RNAs

MicroRNAs (miRNAs) are small non-coding RNAs with 18–24 nucleotides, which play an important role in many biological processes, including regulating gene expression. Long noncoding RNAs (lncRNAs) are RNA molecules larger than 200 nucleotides, which modulate gene expression at transcriptional and translational levels. lncRNAs are RNAs encoded by the human genome that are not translated into proteins (Ratti et al., 2020). The X chromosome contains 10% of all the miRNAs in the human genome, whereas the Y chromosome only contains two miRNAs. The X-linked ACE2 gene is regulated downstream by micro RNAs (miRNAs) and proteolytic cleavage. However, the TMPRSS2 gene is positively regulated by androgens in the prostate and, thus, may demonstrate male-biased expression. The X chromosome encodes major microRNAs such as miRNA-18 and miRNA-19, which play a role in sex differences in immune responses to coronaviruses diseases, including SARS-CoV-2 infection. Other X-linked microRNAs are miR-233 (regulating neutrophil differentiation), miR-106A, miR-424, miR542, and miR-503 (negative regulation of monocyte differentiation), expression of which can be under sex hormone control (Dai et al., 2013; Channappanavar et al., 2017). The high expression of miRNA genes on the X chromosome in females is a result of incomplete X inactivation, which furthers the sex differences in susceptibility to COVID-19 diseases. lncRNAs also play a crucial role in the regulation of innate and adaptive immune responses as catalysts of X inactivation, which leads to sex-differential (Channappanavar et al., 2017).

### Genetic Polymorphisms

Polymorphism in autosomal genes on sex chromosomes encoding immunological proteins can have sex-differential effects on immune responses. It is proposed that epigenetic and hormone-dependent mechanisms might affect sex-based differences in gene variants (SNP) expression, but this needs stronger evidence support (Souyris et al., 2018).

### Sex Hormones

Sex steroids such as estrogen and androgen, as regulators of immune responses, exert their function by binding to estrogen receptors (ER), androgen receptors (AR), and progesterone receptors (PR). These receptors are expressed by immune cells such as B- and T-lymphocytes, macrophages, monocytes, natural killer cells, dendritic cells, and myeloid cells. It has been suggested that androgen receptors may be one of the important factors responsible for gender differences in COVID-19 presentation. As an anti-inflammatory hormone, androgen has a significantly higher expression in men than in women (Chien et al., 2006; Malkin et al., 2004; vom Steeg and Klein., 2016). Androgen in men suppresses pro-inflammatory responses by reducing the secretion of pro-inflammatory cytokines and increasing the production of anti-inflammatory cytokines. An investigation found a higher expression of IL-16, IL-7, ILCs, and IL-18 in males with COVID-19 than in females, which may promote COVID-19-related cytokine storms. Most patients with severe COVID-19 infection have experienced “cytokine storms” that can trigger the immune system to attack the body violently, resulting in an acute respiratory distress syndrome and multiple organ failure. A previous study demonstrated a positive correlation between the severity of pneumonia and the cytokine storm and inflammatory response caused by COVID-19 infection. These findings may hold answers to the question of why the risk of severe COVID-19 and case fatality rate (CFR) in males is higher than in females (Chien et al., 2006; Garanina et al., 2019; Guan et al., 2020b).

### Age

Epidemiologic studies have revealed that individuals older than 60 and those with chronic or preexisting diseases (such as diabetes and hypertension) are more likely to present with severe or lethal forms of COVID-19 infection than younger cases without underlying diseases. This difference is possibly associated with systemic inflammation or cytokine storm (Abdulamir and Hafidh, 2020), considering suboptimal innate and adaptive responses to viral infection in older patients with underlying diseases. It is suggested that differences in immunity response, gene expression level, or even genetic background might explain the variations in susceptibility to SARS-CoV-2 infection, its severity, and mortality rate. Without an adequate immune response, the virus begins to replicate more aggressively, and the deadly critical phase of the disease occurs more frequently and in a shorter time. Severe pneumonia and respiratory failure in COVID-19 infection appear to be the result of an exaggerated immune response and severe inflammation rather than a direct harmful effect of the virus (Yao H.-P. et al., 2020). Another study demonstrated that patients over 60 years have a lower T-cell count, indicating that TNF-α might be directly involved in these patients, leading to an insufficient and weak immune response against viral infection. Children are not immune to COVID-19 and are vulnerable to most of the circulating common coronaviruses. Given that the adults are exposed to many respiratory infections, numerous memory cells are found in adult hosts (Yao H.-P. et al., 2020). However, the reason why COVID-19 does not aggressively attack children as it does for elderly adults is yet unknown. Besides, COVID-19-infected children younger than age 9 show mild signs and symptoms compared to older patients. It is probably due to insufficient memory cells specific to SARS-CoV-2 antigens, leading to a much milder cell-mediated immune response and milder inflammation than what happens in adults. Studies on S-protein RBD and ACE2 demonstrated that a higher binding affinity of RBD to ACE2 leads to higher virus infectivity and pathogenicity. The fact that the RBD of 2019-nCoV exhibits a much stronger affinity to ACE2 in elderly individuals and patients with a weaker immune system or accompanying underlying diseases might be related to a lower level of ACE2 expression in those people, resulting in a high virulence potential for 2019-nCoV (Yao H.-P. et al., 2020).

### Clinical Symptoms and Immunopathology Features

**FIGURE 1:**
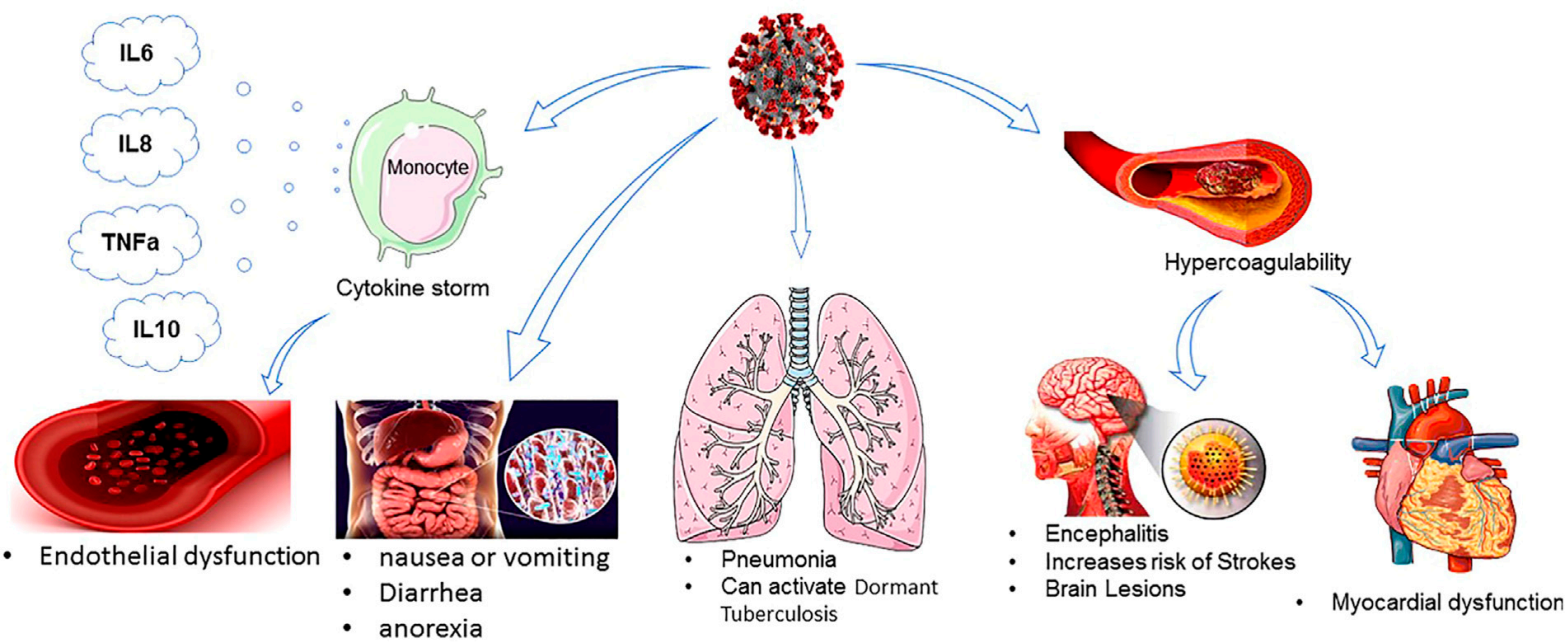
COVID-19 effect on different organisms.

COVID-19 infection can cause mild, moderate, severe, and/or life-threatening pneumonia. The most common symptoms of COVID-19 are fever, cough, running nose, and body ache. These symptoms may develop in different SARS-CoV-2 variants, including Alpha, Mega, Delta, and recently Omicron. In severe cases of COVID-19, loss of smell and taste sensation and difficulty in breathing have been observed, while Omicron-positive people did not have breathing issues to date (Ettaboina et al., 2021). The incubation period of the SARS-CoV-2 infection ranges from 2 to 14 days (Chan J. F.-W. et al., 2020; Guan et al., 2020b; Jiang et al., 2020) compared to the Delta variant of SARS-CoV-2 with a 4-day incubation period. COVID-19 infection may culminate in progressive respiratory failure due to alveolar damage (Ye et al., 2020). The most common manifestation on chest CT scans of the infected patients is peripheral and bilateral ground-glass opacities or consolidative lesions, in which their density and extension have been correlated with disease severity and mortality (Guan et al., 2020a). Patients with mild COVID-19 present with fever, cough, sore throat, fatigue, headache, dyspnea, or myalgia, have normal or decreased leukocyte counts (Peiris et al., 2004), and might show increased blood levels of ALT, AST, LDH, CK-MB, CRP, and ESR (Chan J. F.-W. et al., 2020). Patients with moderate infection often experience dyspnea after 1 week. Patients with severe COVID-19 rapidly progress to critical conditions, including acute respiratory distress syndrome (ARDS), severe pulmonary infection, acute respiratory failure, severe metabolic acidosis and coagulation disorders, hypoxemia unresponsive to conventional oxygen therapy, and septic shock, which can result in multi-organ failure (e.g., acutely altered function in liver and kidneys) and even death. Pathologically, inflammation and immune cell infiltration, necrosis, and hyperplasia are typically inspected in infected tissues and secretions (Guo et al., 2019; Jiang et al., 2020). Damage to the pulmonary interstitial arteriolar walls indicates that inflammatory response is the main determinant of the course of the disease, despite the pathogenic effect of CoVs (Wong C. et al., 2004). Notably, patients with severe forms of infection also had more prominent laboratory abnormalities, including lymphocytopenia, thrombocytopenia, and leukopenia (Guo et al., 2019) (Figure 1). Patients who require intensive care in the course of hospitalization have high levels of pro-inflammatory cytokines including IL-2, IL-7, IL-10, G-CSF, IP-10, MCP-1, MIP-1A, and TNF-α circulating in their bloodstream, which leads to cytokine storm and is highly relevant to disease severity and final clinical outcomes (Huang C. et al., 2020). An early surge in the serum level of pro-inflammatory cytokines can lead to potentially severe disease, similar to what precedes a cytokine storm (Prompetchara et al., 2020; Wu F. et al., 2020). In COVID-19 patients with a higher level of IL-6 and IL-10; increased CRP; elevated levels of ALT, AST, and creatine kinase; increased neutrophils; and decreased lymphocytes, the clinical coarse of disease is more severe and the mortality rate is considerably higher (Huang C. et al., 2020; Wu and Yang, 2020).In contrast, a high level of IL1B, IFNγ, IP10, and MCP1 probably leads to the activation of the T-helper-1 (Th1) cell responses and increases the secretion of T-helper-2 (Th2) cytokines (IL4 and IL10) that suppress inflammation (Mescher et al., 2006). The majority of COVID-19 infected patients had a lower absolute count of lymphocytes. This finding suggests that 2019-nCoV might mainly act on lymphocytes, especially T lymphocytes, as does SARS-CoV. Viral particles cross the respiratory mucosa and infect other cells, induce a cytokine storm in the body, and generate a series of immune responses that, in turn, cause alterations in the number and functionality/activity of peripheral white blood cells such as lymphocytes. Some studies suggest that a substantial decrease in the total number of lymphocytes implies that coronavirus consumes many immune cells and inhibits the cellular immune function of the host. Damage to the T lymphocytes might be an important factor in exacerbating the condition and leading to a rapid clinical deterioration in COVID-19 patients (Chen N. et al., 2020). Moreover, the total count of other immune cells, including CD4+T, CD8+T, dendritic cells (DCs), macrophages, and natural killer cells (NKCs), significantly decreases. What is more, according to previous studies, CD4+T cells, but not CD8+T cells, are important for harnessing the SARS-CoV infection (Bai et al., 2020; Zhu et al., 2010). Nearly 85% of COVID-19 patients are asymptomatic or present with mild symptoms, around 10% may develop with severe symptoms such as the acute respiratory distress syndrome (ARDS), and at least 5% are critical cases who need treatment in the intensive care unit (ICU), of which roughly half die, with an average morality rate of approximately 2.4% (Dandekar and Perlman, 2005; Wu F. et al., 2020). Notably, children and teenagers with COVID-19 infections have milder disease than adults, and fatal cases are more common in elderly patients with chronic and multiple comorbidities, such as hypertension, heart disease, diabetes, endocrine system pathology, digestive system disorders, malignancy, nervous system disease, and respiratory disease, due to weakened immune function (Cai, 2020; Wu C. et al., 2020a; Zhou F. et al., 2020). In an investigation by Guo et al., most of the patients were men, with an average age of 55.5 years. In total, 41.9% of the patients were female, and 0.9% of the patients were younger than 15 years (Guo et al., 2019). Despite the phylogenetic homogeneity between SARS-CoV-2 and SARS-CoVs, it seems that the severity and fatality rate of COVID-19 are lower than those of SARS and MERS (Guo et al., 2019; Li X. et al., 2020a).

## Diagnosis of COVID-19

The detection of the disease at an early stage is essential to isolate COVID-19 patients from healthy people. According to the China guideline of Diagnosis and Treatment of Pneumonitis Caused by SARS-CoV-2 (Corman et al., 2020), available and widely accepted clinical diagnostic tools for COVID-19 include molecular methods of full genome sequencing and real-time quantitative polymerase chain reaction (RT-qPCR)), lung CT scan, serological evaluation of anti-viral immunoglobulin M (IgM), G (IgG) antibodies, and viral culture (Ettaboina et al., 2021). In RT-PCR, respiratory specimens are collected by nasal swab or swab from the throat of an infected patient. This technique is performed by targeting the specific primers in the ORF1ab and N gene regions of SARS-CoV-2. Genome sequencing is almost not applicable given its high cost. Early laboratory assessments mostly consist of complete blood count, blood chemical analysis, coagulation testing, liver function tests, blood urea nitrogen and creatinine, CRP, LDH, ESR, procalcitonin, creatine kinase, and electrolytes (Adams et al., 2020). The low blood count of lymphocytes has also been proposed as a paraclinical indicator to diagnose the COVID-19 infection (Channappanavar et al., 2017). Besides, RT-qPCR also has some limitations, including sample collection and transportation, sample safety, kit performance, and a low positive rate of RT-PCR for throat swab samples (30%–60%) at initial presentation, its false-negative rate, its lack of sensitivity, insufficient stability, and its relatively long processing time (Ai et al., 2020; Pan et al., 2020; Xie et al., 2020). The number of mutations involving epitopes of the SARS-CoV-2 variants (particularly Omicron) has made several single-target molecular tests ineffective, raising the false-negative rate results in patients infected by this VOC. Virus antigens or serological antibody testing kits can be implemented for diagnosis (Channappanavar et al., 2017). Although antigen tests rely mainly on nucleocapsid proteins, they can also detect the proteins of the SARS-CoV-2 Omicron variant but maybe with a lower sensitivity (Tiecco et al., 2022; Ettaboina et al., 2021). The antigen tests are generally less sensitive to very early infections compared with molecular tests. Following the FDA’s long-standing rapid test recommendations, a negative antigenic test in a symptomatic patient or patients with a high likelihood of infection inevitably requires follow-up molecular testing (Tiecco et al., 2022). Over the last few decades, lung ultrasound (LUS) has been a useful, bedside, safe, and non-ionizing imaging modality for diagnosing a variety of acute respiratory diseases, including COVID-19 pneumonia, and it has been superior to chest x-ray and clinical examination (Copetti, 2016; Lichtenstein and Malbrain, 2017). It can be a beneficial diagnostic/triaging tool in emergency departments given resource-limited settings (Lichtenstein and Meziere, 2008). LUS has several advantages over RT-PCR (real-time polymerase chain reaction) as it can precede RT-PCR test positivity (Kalafat et al., 2020), and it is consistent with CT imaging. Moreover, LUS is more sensitive than chest x-ray (plain chest radiography) in both children and adults and in pregnant women without radiation exposure and with ease of sterilization (Pagano et al., 2015; Mayo et al., 2019; Denina et al., 2020; Feng et al., 2020; Huang Y. et al., 2020; Kalafat et al., 2020; Poggiali et al., 2020). Chest CT imaging is a non-invasive, easy-to-perform, and fast method of diagnosis with high diagnostic accuracy and timelier COVID-19 diagnosis compared to RT-PCR, with the only drawback of radiation. It has been reported that almost all COVID-19 patients demonstrate peripherally distributed radiographic features of ground-glass opacity, multifocal organizing pneumonia, and interstitial changes on their CT scan (Ai et al., 2020; Huang P. et al., 2020). Like LUS, chest CT may show pulmonary abnormalities in COVID-19 infected patients with initial negative RT-PCR results (Chung et al., 2020). Individuals who have negative RT-PCR results may benefit greatly from a combination of repeated RT-qPCR tests and chest CT scans (Yang Y. et al., 2020). A low-dose chest CT protocol (mainly based on reducing CT tube current from 100 to 150 mAs in the standard CT protocol to 50 mAs) has been introduced and implemented to overcome the main chest CT scan downside, which does not decrease diagnostic accuracy of CT images but reduces radiation dose considerably (up to 89%) in comparison to the standard-dose protocol (Azadbakht et al., 2021). Regarding some limitations of RT-PCR and CT scans to diagnose COVID-19, immunological detection kits are used to target viral antigens or antibodies in clinical laboratories. SARS-CoV-2 IgG/IgM Antibody Test Kit and ELISA kits for SARS-CoV-2 can detect and quantify SARS-CoV-2 IgM and IgG and are highly sensitive for IgG identification from 10 days following symptoms onset (Adams et al., 2020).

### CRISPR Based Methods and Their Usage in COVID-19 Detection and Treatment

**FIGURE 2:**
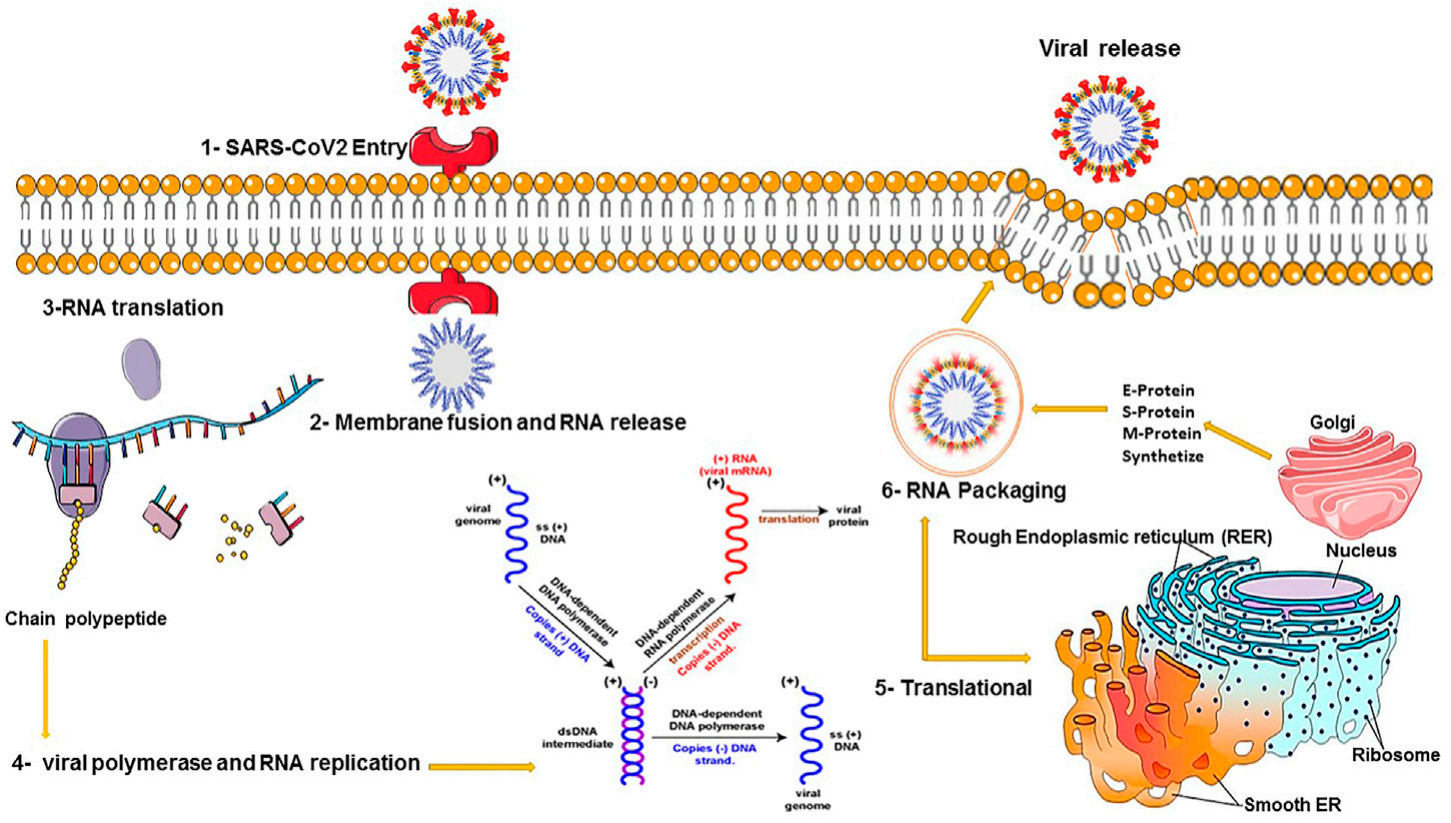
COVID-19 viral replication and translation.

These methods are alternative RNA-based antiviral strategies acting against infections caused by RNA viruses, with the capability of targeting highly different conserved regions by multiple crRNAs, further reducing the chances of viral escape from inhibition through mutation and drug reissuance. Another advantage of this novel strategy is that it targets not only viruses infecting humans but also those that are currently found in animal reservoirs and might transfer to humans unexpectedly. This approach relies on a CRISPR-based system for recognizing and degrading the virus genome or mRNAs. The Cas13a RNA-targeting technique of SHERLOCK (Specific High Sensitivity Enzymatic Reporter Unlocking) as a new efficient CRISPR-based diagnostic test has been developed to rapidly detect all types of RNA viruses. This approach consists of two guide RNAs (gRNAs), which combine with a Cas13 protein and form a SHERLOCK system to detect SARS-CoV-2 RNA in human lung epithelial cells. The gRNAs are designed based on certain specific regions of the S gene and Orf1ab gene in SARS-CoV-2, which can bind to their complementary sequences in the SARS-CoV-2 RNA and recognize the viral RNA. In this system, a paper strip is used for visual readout, similar to a pregnancy test. After dipping a paper strip into a prepared sample, if a new line appears on the strip, it indicates virus existence in the sample. As it takes only 45 min to detect viral RNA in the sample, the new test works faster than widely used RT-PCR techniques, taking about 4 hours to reveal the result in a respiratory sample (Curti et al., 2020). DNA Endonuclease-Targeted CRISPR Trans Reporter (DETECTR) is another low-cost and accurate CRISPR-Cas12-based approach developed for SARS-CoV-2 detection in respiratory samples in approximately 30 min. Cas12 gRNAs are designed to specifically detect the N gene in SARS-CoV-2 and the E (envelope) and the N (nucleoprotein) genes in other viral strains (Freije et al., 2019; Mustafa and Makhawi, 2020). Moreover, two novel CRISPR-Cas13 techniques, CARVER (Cas13-assisted restriction of viral expression and readout) and PAC-MAN (Prophylactic Antiviral CRISPR in Human Cells), have been developed for therapeutic purposes, which target the SARS-CoV-2 virus and restrict its replication (Figure 2).

**FIGURE 3:**
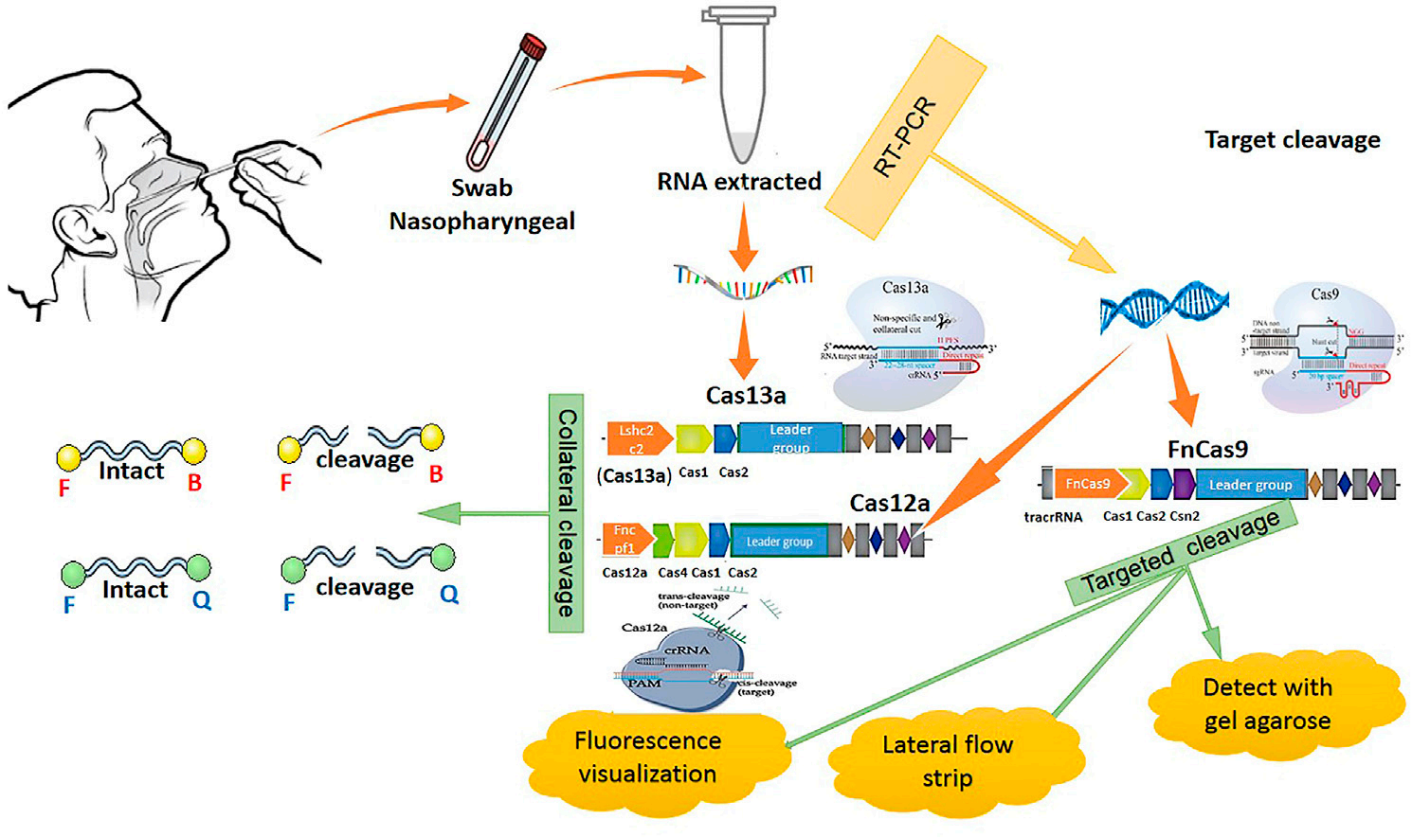
CRISPR methods for COVID-19 detection.

In order to clear the virus, these techniques contain guide RNAs (gRNAs) designed to organize the viral RNA by specifically binding to complementary sequences in the viral RNA genome and effectively cut it using an RNA-targeting Cas13 nuclease (Konermann et al., 2018). Guide RNAs have been designed based on highly conserved regions of the viral genome encoding the major structural proteins of SARS-CoV-2, including the orf1ab, S (spike), M (membrane), N (nucleocapsid), and E (envelope) genes. Targeted inhibition of these proteins can lead to disabled viral replication and function. A total of 10,333 guide RNAs have been designed to specifically target 10 peptide-coding sites on the RNA genome of SARS-CoV-2. This novel approach is applicable to defend against the virus variants that evolve and may escape traditional drugs and vaccines. It particularly targets the different regions of the same virus or different SARS-CoV-2 strains (L and S strains) by the crRNAs pool (Konermann et al., 2018). Cas13d and its components can be delivered within polymers or lipid nanoparticles with chemical alterations to increase stability. Besides, a recently developed DNA-based liposomal delivery strategy (i.e., the HEDGES platform) is also optimal in this regard (Konermann et al., 2018). If we find this therapeutic strategy secure and effective with a safe delivery method, it could be a good alternative to traditional vaccines in which viruses can escape from inhibition. Among different virus vectors, the adeno-associated virus (AAV) can be used to deliver the CRISPR/Cas13d system to infected lung cells in SARS-CoV-2 patients (Hoffmann et al., 2020b). Before *in vivo* therapeutic application of the CRISPR/Cas13d system on patients, it is necessary to determine the safety and efficacy of this system in clearing 2019-nCov and other viruses in animals (Broughton et al., 2020; Cui et al., 2019) (Figure 3).

### Innate Immune Responses Mechanism to SARS-CoV-2 Infection

The epithelium of the lungs and the respiratory tract are exposed to viruses existing in the inhaled air. SARS-CoV-2 entry into the respiratory tract cells depends on its attachment to the ACE2 receptor on the surface of the host’s lung cells by the viral spike (S) protein, leading to antigen presentation *via* APCs. Antigen detection by the innate immune sensors in the respiratory tract stimulates humoral and cellular immunity, mediated by virus-specific B and T cells. Indeed, the appearance of viral dsRNA in the cytoplasm and its detection by pattern recognition receptors (PRRs) [including the Toll-like receptors (TLR3 and TLR7), cytotoxic T lymphocytes (CTLs), cytosolic RNA sensor, and RIG-I/MDA5], triggers the downstream signaling cascade of NF-κB and IRF3 against RNA viruses in the lungs (Totura et al., 2015; Zhou et al., 2014). Alveolar lymphocytes and macrophages, dendritic cells (DCs), airway epithelial cells, and innate lymphoid cells as innate immune contributors induce the secretion of large amounts of cytokines and chemokines, establishing an antiviral state in the lung cells (Taniguchi and Takaoka, 2001). This antiviral state stimulates the production of type I and type III interferons (IFNs), genes (ISGs), and other proinflammatory cytokines (TNF-α and IL-6), which control viral replication by inducing an effective adaptive immune response. Furthermore, type I IFN simulates the release of antiviral proteins to protect neighboring uninfected cells (Trottein and Paget, 2018; Zhou et al., 2018). In general, T helper cells, especially Th17 cells, produce the proinflammatory cytokine IL17 *via* the STAT3 and NF-κB signaling pathways and assist cytotoxic T cells and B cells to clear the virus. Natural killer T cells, mucosal-associated invariant T cells, and neutrophils also effectively mediate the innate and adaptive immune responses, clearing viruses from the respiratory system (Martin and Frevert, 2005; Gabay, 2006). All the aforementioned immune responses can prompt an uncontrolled immune-inflammatory response called a “cytokine storm.” The cytokine storm denotes highly increased levels of pro-inflammatory cytokines (IFN-α, IFN-γ, G-CSF, IL-1b, IL-2, IL-6, IL-7, IL-10, IL-12, IL-18, IL-33, TNF-α, TGFb, MCP-1, MIP-1A, etc.) and chemokines (CCL2, CCL3, CCL5, CXCL8, CXCL9, CXCL10, etc.) in severe SARS-CoV-2 infection (Kenway-Lynch et al., 2014; Jones and Jenkins, 2018). The serum levels of TNF-α, IL-6, and IL-10 in SARS-CoV-2-infected patients are negatively correlated to the total number of T cells, CD4+T, and CD8+T cells, suggesting that cytokine storm might reduce T-cell count *via* apoptosis or necrosis (Williams and Chambers, 2014). Moreover, high initial viral loads, and an increased number of IMMs and neutrophils in the lungs, accompanied by elevated pro-inflammatory cytokine/chemokine levels, cause lung damage in SARS patients (Xu Z. et al., 2020). Acute respiratory distress syndrome (ARDS) and multiple organ failure, which are the main death causes in COVID-19 infections, are results of the cytokine storm following severe pulmonary inflammation (Cameron et al., 2008; Sui et al., 2008). Humoral immunity (B cells), as the adaptive immune response, defends only against viruses outside the cell (i.e., extracellular viral particles). In fact, neutralizing antibodies bind to the crown-like spikes of these extracellular viruses and prevent them from interacting with host cells and even prevent re-infection in the future. Incomplete, delayed, or even strongly induced host immune response can result in pulmonary tissue damage (Ter Meulen et al., 2006; Kikkert, 2020). The adaptive immune evasion mechanism is a primary viral reaction to escape host immune detection and suppress innate immune responses aimed at lengthening the virus’s survival, boosting its replication rate, and promoting the host’s cell infection (Prompetchara et al., 2020). For this purpose, SARS-CoV-2 can downregulate MHC class I, MHC class II, and CD80/86 in antigen-presenting cells (APCs) by infecting macrophages or dendritic cells, which inhibit T-cell activation. SARS-CoV-2 also modifies intracellular membranes to form double-membrane vesicles that lack PRRs, thereby replicating in these vesicles and avoiding the host detecting their dsRNA (Lessler et al., 2009; Liu et al., 2018; Snijder et al., 2006). This is probably associated with a longer incubation period of 2–14 days in SARS-CoV-2 compared to other CoVs (Martin and Frevert, 2005). Besides, some accessory proteins of SARS-CoV-2 can attach to the dsRNA during replication to block the TLR-3 activation and evade the immune response. Another viral strategy for suppressing the host immune response is encoding viral proteases for cleaving innate immune factors (Martin and Frevert, 2005). This interaction between the cellular immune responses and viral evasion mechanisms identifies infection outcomes (Gralinski and Baric, 2015; Farrag and Almajhdi, 2016). Moreover, delayed innate immune response that is secondary to temporary suppression when an innate immune response has been evaded contributes to an overreaction and exacerbated immune response, which leads to cytokine storms, damaging inflammation, and other severe complications (Røsjø et al., 2011; Farrag and Almajhdi, 2016). However, in-depth knowledge of this virus–host interaction proposes important opportunities for designing and creating novel antiviral strategies. Because each virus uses various strategies for suppressing the host immune response *via* evolved multifunctional proteins, mostly it is difficult to determine the immune evasive pattern to offer novel antiviral treatment strategies based on it. Notably, long-lasting immune protection after SARS-CoVs infection has not yet been proved, and indeed, CoVs can re-infect individuals sometime after earlier infection. It might be associated with the virus’s high genetic variation and genetic drift and shift, causing new strains that are not efficiently recognized by the innate immunity system. This is the reason why individuals experience various CoV infections in the course of their lives (Gralinski and Baric, 2015).

### Genetic Variability in Immune System and Susceptibility to COVID-19

Recently, a study investigated the probable relationship between the global distributions of three immune system genes, called human leukocyte antigen (HLA) A, B, and C genes, involved in recognizing pathogens, with potential epidemiological outcomes of the current pandemic. This investigation revealed that the effect of genetic variation in the human immune system genes (HLA), as significant segments of the viral antigen presentation pathway, may affect an individual’s susceptibility to COVID-19 and the severity of infection. It has been suggested that genotype variability in HLA genes causes differences in the capability of recognizing a pathogen and affects the capacity of the immune response against COVID-19 infection. Thus, certain alleles might be responsible for more susceptibility and more severe infection in some individuals. Because of poor recognition of SARS-CoV-2 by this haplotype, some patients may be more vulnerable to the virus. It has been demonstrated that individuals with the HLA-B*46:01 allele and the fewest binding peptides for SARS-CoV-2 show a particular vulnerability to COVID-19, as previously shown for SARS. Using this knowledge, individuals with high-risk HLA types could be prioritized in vaccine design against SARS-CoV-2 (Cui et al., 2019).

### Viral Transmission and Spread

SARS-CoV-2 was initially zoonotic in nature and then gained the potential for human-to-human transmission. The source of infection for this novel virus is mainly a virus-contaminated hand touching the mouth and nose (Jiang et al., 2020). The most important route of transmission for SARS-CoV-2 is person-to-person transmission through close contact with infected patients or respiratory droplets, which they spray when coughing, talking loudly, or sneezing (Phan et al., 2020; Rothe et al., 2020). Vertical transmission of the novel coronavirus or transmission through breastfeeding has not yet been proven (Rothe et al., 2020). COVID-19 is most contagious during the asymptomatic incubation period (roughly between 2 and 14 days) (Lu C.-w. et al., 2020). Viral load and the severity of the disease are the main determinants of inter-host transmission. However, there is no direct evidence for the transmissibility of coronaviruses from contaminated surfaces to hands (Cao et al., 2020). Although early reports were supportive of the absence of human-to-human transmission for the virus, it is currently clear that efficient human-to-human transmission is essential for the large-scale spread of 2019-nCoV. The basic reproductive number (R0) of COVID-19, the number of individuals that each case can infect, determines its transmissibility, ranging from 2.2 to 2.6 (Li X. et al., 2020b). Given that 2019-nCoV uses the human angiotensin-converting enzyme 2 (hACE2) as a host receptor, it is likely that 2019-nCoV will further adapt to the human host by increasing its binding affinity to hACE2. Adaptation to the human host depends on some mutations that frequently occur during viral RNA replication errors or through recombination events in SARS-CoV-2, which support their tendency for human infection (Ge et al., 2013; Chen J et al., 2020). The viral mutations mostly occur in the receptor-binding motif (RBM) of the SARS-CoV S-protein and increase the binding affinity of the viral spike protein to the human cell receptor ACE2. These mutations facilitate the virus’s replication and assist the virus in adapting to the new conditions in the human host cell. Consequently, the new virus could undergo adaptive evolution, which results in more efficient human-to-human transmission and can increase the virus’s virulence. In fact, interactions between the receptor-binding domain (RBD) of the SARS-CoV-2 spike protein and its host receptor, angiotensin-converting enzyme 2 (ACE2), mediate both the cross-species and human-to-human transmissions (Chan J. F.-W. et al., 2020; Liu Z. et al., 2020). Among different SARS-CoV-2 variants, Omicron shows the highest transmissibility. It might be due to multiple mutations in the virus genome that increase the binding affinity to the ACE2 receptor. For instance, N501Y mutation combined with Q498R, E484K, S477N, and H69/V70 deletions might increase the binding affinity to ACE2 receptor by up to 1000-fold and up to the level of low pM in KD value. The existence of H655Y and N679K mutations in the furin cleavage site is supposed to enhance spike cleavage (S1/S2 junction) and lead to more transmission. Moreover, the P681H mutation Alpha variant and P681R in the Delta VOC probably increase the transmission rate through the same mechanism. Other Omicron spike mutations, including K417N, G446S, E484A, Q493R, G496S, and Y505H, might also impact virus transmissibility *via* increasing binding affinity to human ACE2. In contrast, RBD mutations such as E484A, Y145del, and Y505H may result in the complete loss of interactions between an antibody and RBD, raising immune escape and reinfections (Tiecco et al., 2022). In the case of COVID-19, it is suggested that one or more mutations may be selected and sustained during the SARS-CoV-2 outbreak as the virus adapts to human hosts, which possibly reduces the virus’s ability to infect cells and might have influenced its transmissibility (Anwar et al., 2013). Moreover, the currently reported 17 non-synonymous mutations that happened in the ORF1ab, ORF7a, ORF8, and spike genes of the SARS-CoV-2 genome (Tortorici et al., 2019) in a family cluster of COVID-19 patients supported the hypothesis that the viral mutations could have occurred during person-to-person transmission. Indeed, due to positive selection pressure, these mutations can enhance the transmission of this novel virus to the new host(s) for its survival (Anwar et al., 2013).

### Treatment Strategies Against SARS-CoV-2

Since the beginning of the new pandemic, many clinical trials have been conducted and numerous specific drugs have been utilized to treat COVID-19 infection and prevent mortality. However, several drugs that were documented to be useful in small clinical trials were ineffective in larger studies. Current COVID-19 treatment options, proved or authorized by the FDA, include antiviral drugs (molnupiravir, nirmatrelvir, and remdesivir), anti-SARS-CoV-2 monoclonal antibodies (bamlanivimab-etesevimab, casirivimab-imdevimab, and sotrovimab), anti-inflammatory drugs (dexamethasone), and immunomodulator agents (baricitinib, tocilizumab) (Takashita et al., 2022). Despite the success of some SARS-CoV-2 therapeutic strategies, such as effective vaccines, COVID-19 treatment remains challenging due to the large number of mutations that have continuously emerged in the SARS-CoV-2 variants and may contribute to attenuating the effectiveness of current treatments. Remdesivir was the first FDA-approved drug in COVID-19 treatment. To date, only the antiviral remdesivir has been shown to facilitate significant clinical improvements and has been approved by major drug safety regulators for COVID-19 (Takashita et al., 2022). Supportive treatment consists of oxygen therapy, invasive mechanical ventilation, membrane oxygenation, prescribing systemic glucocorticoids, and renal replacement therapy (CRRT) (Lu L. et al., 2013). Oxygen therapy is used for patients with a severe acute respiratory infection, respiratory distress, hypoxemia, or shock (Schultz et al., 2017; Bai et al., 2020). For early treatment for fever, antipyretic therapies are administered (e.g., paracetamol), and guaifenesin would be helpful for non-productive cough (Liu K. et al., 2020). Intravenous administration of immunoglobulin and steroids (methylprednisolone) can enhance the anti-infection status in critical patients with ARDS and septic shock, who otherwise may experience multiple organ failures. In the absence of shock, intravenous fluids should be carefully administered (Channappanavar et al., 2017). Renal replacement therapy (RRT) is an option for patients with AKI, and antibiotics are the mainstay of therapy in sepsis (Li and De Clercq, 2020). Antiviral RNA-target therapy, antibody and plasma therapy, immunomodulatory therapy, oligonucleotide-based therapies, peptides and interferon therapies, traditional Chinese therapies, and vaccines are also used to treat 2019-nCoV infection (Coleman et al., 2014; Lu, 2020). SARS-CoV-2 components might serve as potential targets for developing antiviral drugs and vaccines, particularly spike protein (S), serine protease TMPRSS2, polymerases, and ACE2 receptors, which are mainly involved in the virus’s entry into the host cell and viral replication (Wu C. et al., 2020b). Because the SARS-CoV-2 spike protein has a higher binding affinity to ACE2 than the spike proteins of other SARS-CoVs, it may be an ideal target for neutralizing antibodies and vaccines designed to combat viral infection. Anti-SARS-CoV antibody functionally intervenes with a special feature of the coronavirus (i.e., viral S-protein-receptor attachment) (Yang et al., 2013). Structural proteins, membrane proteins, and other accessory proteins of SARS-CoV-2 (ORFs, RBD) can also make credible vaccine targets (Li Y. et al., 2020). Specific inhibitors targeting key proteases such as TMPRSS2 (involved in replication and proliferation of the virus) are among the most effective drugs for treating COVID-19 infection (Xu H. et al., 2020). ACE2, as a receptor protein in both SARS-CoV and 2019-nCoV, is highly expressed in the epithelia of the human lung and small intestine, and coronavirus can infect human cells *via* binding to this receptor (Kawase et al., 2012). Thus, ACE2 can be considered an ideal target for 2019-nCoV infection treatment. Camostat mesylate is an approved serine protease TMPRSS2 inhibitor used in Japan for the off-label treatment of SARS-CoV-2 infected patients (Sheahan et al., 2020).

### Antiviral Therapy

Antiviral therapy against SARS-CoV-2 consists of many of the medications previously prescribed for SARS/MERS, influenza, and HIV infections, including nucleoside analogs (lopinavir, ribavirin, chloroquine, remdesivir, ritonavir, favipiravir, and galidesivir), neuraminidase inhibitors (oseltamivir), spike protein inhibitors (griffithsin), RNA synthesis inhibitors (TDF, 3TC), anti-inflammatory drugs, fusion peptide (EK1), abidol, and Chinese traditional medicine (ShuFengJieDu capsules and Lianhuaqingwen capsule) (Savarino et al., 2006; Zumla et al., 2016; Ji S. et al., 2020; Wang M. et al., 2020). However, the efficacy and safety of some of these drugs in COVID-19 treatment have not been confirmed. Hydroxychloroquine, which was used to prevent COVID-19, had no effect on preventing illness, hospitalization, or death from the disease and may increase the risk of cardiac arrhythmia, blood and lymph disorders, kidney injury, and liver problems. Based on previous evidence, ribavirin and lopinavir-ritonavir have been effective for HIV, SARS, and MERS infection therapy. The clinical application of two HIV-1 protease inhibitors, namely, lopinavir and ritonavir, appears to be effective in suppressing COVID-19 infection (Wang et al., 2021). Remdesivir, an inhibitor of the viral RNA-dependent RNA polymerase (RdRp), has a similar chemical structure to HIV reverse-transcriptase inhibitors. It targets the RNA-dependent RNA polymerase and inhibits viral RNA synthesis in human coronaviruses. Remdesivir, originally used to treat Ebola, is an effective and nearly safe treatment for COVID-19 infection (Savarino et al., 2006; Richardson et al., 2020). A recent study suggested the various mutations in the RdRp (V557L, V473F, N491S, F480 L/S/C, P323L, and or E802D) are associated with remdesivir resistance in the Omicron SARS-CoV-2 variant (Chen et al., 2022)*.* A combination of lopinavir/ritonavir or ribavirin as nucleoside analogs with IFNs as protease inhibitors can act synergistically upon 2019-nCoV and reduce the mortality rate in critical conditions (Ji S. et al., 2020; Wang M. et al., 2020). The antiviral drug nirmatrelvir (PF-07321332), co-administered with ritonavir, inhibits the SARS-CoV-2 protease involved in viral replication. Molnupiravir, used orally for patients with mild-to-moderate COVID-19, increases the frequency of viral RNA mutations and impairs SARS-CoV-2 replication. Molnupiravir is expected to be effective against all SARS-CoV-2 variants, including the recently discovered Omicron (Jayk Bernal et al., 2022). Several approved antiviral drugs such as nafamostat, camostat, or aprotinin have been effective in SARS-CoV-2 variants, especially Omicron. The Pfizer company confirmed that the nirmatrelvir pill was effective in patients infected with Omicron (Ettaboina et al., 2021).

### Antibody and Plasma Therapy

Neutralizing monoclonal antibodies include sotrovimab (GSK4182136 or S309), bebtelovimab (LY-CoV1404), bamlanivimab-etesevimab (LY-CoV016-LY-CoV555), and casirivimab-imdevimab (REGN-CoV2), which prevent infection of human cells by blocking the S-protein–ACE2 attachment and mediate SARS-CoV-2 entry into the human respiratory epithelial cells (Tiecco G et al., 2022). These antibodies target the spike protein (S), act as antigenic proteins, and stimulate the antibody response by interacting with the cell surface receptor angiotensin-converting enzyme 2 (ACE2). Long-term antibody response is affected by antibody concentration and the severity of the infection (Liu and Wang, 2020; Tian et al., 2020). Bamlanivimab-etesevimab (LY-CoV016-LY-CoV555) is a cocktail of two antibodies binding to the RBD of the SARS-CoV-2 S-protein, blocking the virus attachment to the human ACE2 receptor, and thereby increasing the neutralization rate. Casirivimab-imdevimab (REGN-CoV2) also reduces the risk of severe COVID-19 infection by binding to distinct epitopes of the S-protein. The neutralization potency of both cocktails is still considered a treatment option for Delta VOC but is greatly reduced in the Omicron variant (Quiros-Roldan et al., 2021). Recombinant human monoclonal antibodies (mAbs), including CR3022, m396, and CR3014, can also neutralize viral function *via* binding to the receptor-binding domain (RBD) of SARS-CoV-2 (Gierer et al., 2013; Nguyen et al., 2020). It is notable that the receptor-binding domain (RBD) on the spike protein is a critical target for neutralizing antibodies. In contrast to the Beta or Delta SARS-CoV-2 variants, the Omicron variant has a significant ability to escape humoral immunity and neutralizing antibodies due to 15 mutations (such as E484A, K417N, D614G, N501Y, K417N, and P681H) identified in antigenic sites (RBS-A, RBS-B, RBS-C, the CR302, and S309) on its spike RBD. These mutations result in increased ACE2 binding affinity, increased transmissibility and pathogenicity, potential resistance to monoclonal antibodies targeting these sites, and immune evasion (Yuan et al., 2021). Besides, both Beta and Gamma variants could escape the neutralizing antibodies of LY-CoV555 due to the E484K mutation in spike and LY-CoV016 due to the K417N/T mutation (Starr et al., 2021). Because CoVs are very potent in generating antibody-escape mutations in the RBD, antibodies that target non-RBD epitopes are wiser choices for COVID-19 treatment (Sui et al., 2008; Shimojima et al., 2015).

### Immunomodulatory Therapy With Anti-Inflammatory Drugs

Cytokine storms (CRS) are the result of the high activation of immune cells and inflammatory responses. Many investigations have reported high levels of pro-inflammatory cytokines and chemokines, including IL-2, IL-6, IL-10, IFN-γ, CXCL10, and CCL2 in COVID-19. Interleukin-6 (IL-6) is a strong pro-inflammatory cytokine that plays an important role in the body’s fight against viral infection (Hertanto et al., 2021; Villaescusa et al., 2022). IL-6 is produced by several cell types, such as T cells, B cells, monocytes, and fibroblasts. TNF-α and IL-1b activate IL-6 expression, which has a key role in the mechanism of acute inflammation. According to the findings of clinical trials, both immunosuppression (IL-1 and IL-6 inhibitors) and immunomodulation (interferon alpha (IFN-α) and interferon beta (IFN-β)) and convalescent plasma therapy have been proposed as an effective treatment only in severe or critical COVID-19 conditions who require respiratory support and ventilation. Anti-inflammatory drugs that inhibit the IL-6 pathway include sarilumab and tocilizumab, which block the IL-6 receptor, siltuximab and lizumab, which block IL-6 itself. Moreover, IFN-β-1a, IFN-α-2b, chloroquine, lopinavir, ritonavir, atazanavir, and ritonavir prevent mortality by reducing inflammation *via* viral clearance from the upper respiratory tract (Hertanto et al., 2021; Villaescusa et al., 2022). Interleukin-10 plays an immunomodulatory role by affecting T-helper 1 and inhibiting IFN-γ, IL-12, and TNF-α. This might result in a reduction of the damaging effects of inflammatory pneumonia (which is a critical phase in COVID-19 pathophysiology). Interferons (IFNs) are cytokines made and released by host cells in response to viral pathogens. A combination of interferons (IFNs) and ribavirin is more effective against coronaviruses compared to the IFNs alone (Morgenstern et al., 2005; Tian et al., 2020). Furthermore, SARS-CoV-2 can escape innate immunity early during the infection by inhibiting IFN effects in viral replication and spread. Currently, corticosteroids (dexamethasone, hydrocortisone, and prednisone) with potent anti-inflammatory effects have been used to treat only severe or critical COVID-19, according to WHO recommendations. However, the role of immune system modulation in SARS-CoV-2 variants (Alpha to Omicron) remains unknown.

### Vaccine

To date, the vaccine design for coronaviruses has faced many challenges. Due to the high rate of genetic recombination and mutation in SARS-CoV-2 variants, developing a suboptimal vaccine may potentially increase the evolution and diversity of the virus in the wild. Despite this, several strategies are being considered for vaccine development to reduce the likelihood of recombination (Prompetchara et al., 2020). In general, it is assumed that live attenuated vaccines must either induce stronger immune responses than the original virus or diminish the disease during secondary mucosal infection. In this regard, for developing vaccines, spike (S) and its receptor-binding domain (RDB) might be considered as an antigen protein, which can stimulate the production of neutralizing antibodies to block the entry of the virus into the host cells (Pardi et al., 2018). According to disruptive vaccine technology, nucleic acid-based vaccines (including RNA or mRNA vaccines) can be designed to provoke an immune response against COVID-19 infection and can be delivered by lipid nanoparticles or an LNP mechanism. Reverse genetic technology can be used to develop new vaccines to combat COVID-19 by utilizing the virus’s innate immune evasive function (Maruggi et al., 2019; Ramaiah and Arumugaswami, 2020). The attenuated virus may trigger better innate immune responses due to the lack of one or more of its evasive functions. Experimental SARS vaccines, including recombinant S-protein and inactivated viruses, induce neutralizing antibodies to raise against SARS-CoV and can offer some protection against SARS-CoV-2 infection (Yang et al., 2004; Wang C. et al., 2020). Current COVID-19 vaccines validated for use by WHO include Pfizer–BioNTech-BNT162b2, Moderna COVID-19 vaccine (mRNA 1273), SII/COVISHIELD and AstraZeneca/AZD1222 vaccine, Sinopharm, Sinovac-CoronaVac, Covovax (NVX-CoV2373) vaccine, and Bharat Biotech BBV152. These vaccines are based on different platforms, including DNA plasmid-based, RNA platform, innovative nucleoside-modified viral messenger RNA encapsulated within nanoparticles, specifically lipid ones (LNPs), non-replicating viral vector, inactivated or weakened versions of the virus, and protein subunit platform (Tsiambas et al., 2021). Recent evidence suggests that the current COVID-19 vaccines might be less effective against the Omicron variant in comparison to other SARS-CoV-2 variants. That is why even three doses of mRNA vaccines may not be sufficient to prevent infection and symptomatic disease with this VOC. This is due to an elevated number of mutations in the Omicron N-terminal domain (NTD) and RDB, significantly making them difficult to be recognized by the NTD/RDB targeting neutralizing antibodies, allowing reinfection, and reducing the efficacy of currently used vaccine (Liu, L et al., 2021)*.* Other observations revealed the importance of variant-specific vaccines based on the mutated spike, especially against the Omicron variant. However, the two-dose Pfizer vaccine had a high level of neutralizing power against other variations, such as Delta, which have been proven to help reduce disease severity, hospitalizations, and death (Ettaboina et al., 2021).

### Psychological Impact of COVID-19 Pandemic

Since the emergence of SARS-CoV-2, continuing COVID-19 crises such as experiencing the new waves of infections caused by different SARS-CoV-2 variants (Alpha to Omicron), viral reinfection, vaccination delay and the non-existence of potent pharmacological treatments for COVID-19, and pandemic fatigue eventually have led to psychological exhaustions, which negatively affects the adherence to health protocols. The COVID-19 epidemic has spread fear, anxiety, depression, and even panic in the community, leading to relapses or worsening of the pre-existing psychiatric disorders in patients because of their higher susceptibility to stress in comparison to the general population (Yao H. et al., 2020). Mental disorders are associated with increased susceptibility to infections and can enhance the risk of infections, including pneumonia. The psychiatric illness itself may also result in lower COVID-19 treatment efficacy. Therefore, clear information on the role of mental disorders in COVID-19 transmission, infection, and the mental health status of the community is urgently required (Santos, 2020). Research conducted on previous infections such as SARS/MERS-CoVs and Ebola has revealed their vast psychosocial impacts on individuals, communities, and international levels during infection outbreaks (Yao H. et al., 2020). Although, currently, there is no detailed data on the psychological impact of the COVID-19 pandemic on the public health while it hit its peak, some pieces of evidence show the impact of the COVID-19 pandemic with anxiety disorders, especially cases of obsessive-compulsive disorder (OCD) who suffer from continuous hand washing immediately after coughing, sneezing, or after touching contaminated objects. Long-lasting perceived stress resulting from COVID-19 can lead to severe OCD symptoms for those who are overanxious about the virus (Zhang and Ma, 2020). Research has also reported that vast, negative, and extended psychological effects such as anger, confusion, and post-traumatic stress symptoms may be associated with long-lasting quarantine, fear of infection, frustration, a lack of basic and needed supplies, misinformation, and financial problems (Zhang and Ma, 2020). Many COVID-19 patients with mental disorders have not been able to get regular health services, and many medical appointments have been suspended. This results in an increased risk of psychiatric disorders, creates a new set of challenges in treatment, and can potentially make therapeutic measures less effective (Santos, 2020). Also, healthcare professionals and many medical practitioners, as the first line of defense against COVID-19, experience severe depression, anxiety, emotional distress, burnout, and post-traumatic stress disorder (PTSD) at the onset, during, and after the end of the outbreak of such infections because of the extreme workloads, physical and mental exhaustion, insomnia, anxiety, and the fear of being infected or transmitting the infection to their families while doing their job with little or substandard protective equipment (Zhang and Ma, 2020). However, several psychiatric comorbidities have been observed even among non-infected people during the COVID-19 pandemic, including depression, panic attacks, anxiety, cognitive distress, psychomotor excitement, suicidality, delirium, and post-trauma stress symptoms due to the fear of getting exposed to COVID-19 (Ayittey et al., 2020; Santos, 2020). Symptoms of the viral infection, such as cough and fever, may also be associated with the emergence or worsening of cognitive distress and anxiety. In a survey study, the psychological impact of the COVID-19 outbreak on mental health status was measured using the Depression, Anxiety, and Stress Scale (DASS-21) and IES-R scale. The results revealed that the COVID-19 pandemic could trigger the onset of the mental symptoms of depression, moderate-to-severe anxiety, insomnia, and distress among the physicians and nurses, caretakers, and young or old affected people (Ayittey et al., 2020). In a study investigating the psychological impact of the infection outbreak, a significant correlation was found between the female gender, student status, and specific physical symptoms such as myalgia, dizziness, coryza, and poor health status with higher levels of stress, anxiety, and depression (Ding et al., 2017). The prevalence of moderate or severe psychological impact, as measured by IES-R, was higher than depression, anxiety, and stress, as measured by the DASS-21. Most respondents (>70%) were worried about their family members contracting COVID-19. Sociodemographic data suggest that females are more affected by the psychological impact of the outbreak (i.e., they suffer from higher levels of stress, anxiety, and depression). This finding was in line with previous exhaustive epidemiological studies, which found that women were at a higher risk of mental disorders such as depression (Ding et al., 2017). Furthermore, the uncertainty of the COVID-19 status could have an adverse effect on the mental health of students and their educational status. Some studies revealed that the general population experienced the negative psychological impact of the COVID-19 outbreak to a higher degree. This also correlates to the high rate of older adult suicide deaths reported in Hong Kong among the affected individuals during the SARS and MERS epidemics (Cui et al., 2019). A study conducted on the psychological impact of SARS infection on affected families showed that they were socially isolated even after being treated and disease-free because the public avoided them (Ayittey et al., 2020). However, the clear and up-to-date information on medicines and treatment plans, routes of transmission, health status and number of both infected cases and recovered individuals, and involved families provided for the medical staff and general public is associated with decreased COVID-19 related psychological impact and levels of stress, anxiety, and depression (Ayittey et al., 2020; Zhang and Ma, 2020). Generally, behavioral therapy based on the model of stress adaptation coupled with psychotherapeutic treatments should also be provided for people with signs of mental disorders to reduce the cognitive effects of the pandemic. Using masks and medical gloves, regardless of the presence or absence of infection symptoms, is associated with less degrees of anxiety and depression. Psychological first aid (PFA) or psychosocial support is helpful in decreasing the acute distress and focuses on the mental health of the affected survivors and clinicians to offer them safety, comfort, and practical help and help them meet their needs after traumatic events such as the COVID-19 pandemic (Ayittey et al., 2020).

## Economic Impacts of COVID-19 on China and the World

The impact of the recent COVID-19 outbreak on the world economy has become a matter of increasing concern. Governments are struggling to prevent the spread of the COVID-19 infection, contain the pandemic, and diminish the adverse effects of the pandemic on trade between nations. Uncertainties about the COVID-19 pathophysiology and its causative pathogen have interrupted global trade and supply chains, decreased asset prices, and forced international businesses to make hard decisions (Atkeson, 2020; Fernandes, 2020; McKibbin and Fernando, 2020). China’s position as the world’s largest manufacturer and importer of crude oil has caused economists to ignore their prediction of yearly global growth, especially in the stock market (Fernandes, 2020). During the SARS-CoV outbreak in China from 2002 to 2003, the global economy nearly lost roughly $40 billion. The current Chinese economy is several times larger than during the SARS epidemic and is even more connected to the whole world. This integration of China, the world’s second-largest economy after the United States, with the rest of the world has resulted in a significant impact of COVID-19 on the global economy. As China now contributes to approximately 16.3% of the world’s GDP, the country has been the main growth driver worldwide, with IMF estimating that China alone accounted for 39% of the global economic growth in 2019 (McKibbin and Fernando, 2020). This implies that any deceleration in the China economy could likely affect the global economy. Some economists, however, estimate that, in the event of the continuous spread of the Wuhan 2019-nCoV, China is expected to lose up to $62 billion in the first quarter of 2021, while the world is likely to lose over $280 billion in the same period. Nevertheless, some economists believe that it is too early to estimate the total impact of COVID-19 on the world’s economy and trade since the infection has not yet reached its peak. According to an estimated model by the same experts, the global GDP is likely to decline by roughly 0.42% in the first quarter of 2021 due to the outbreak. Furthermore, the negative impact of the crisis will be greater on the service-oriented economies, which means more jobs are at risk. Countries whose economies are highly dependent on tourism (more than 15% of GDP), such as Greece, Portugal, and Spain, will be more negatively influenced by this crisis. This current crisis is posing adverse effects on countries that are highly dependent on foreign trade by affecting supply chains. The literature suggests that if the crisis continues, it will cost 2.5%–3% of global GDP per month on average (McKibbin and Fernando, 2020).

## Conclusion

The continuous emergence of new SARS-CoV-2 variants has made the control of the COVID-19 pandemic more complicated. It is also not clearly known whether new variants evolving after the Omicron variant will bear a higher transmission rate, infection capacity, and immune-escape potential. However, there is no doubt that the Omicron variant will not be the last variant of SARS-CoV-2. Fortunately, we have accumulated a lot of experience and methods to deal with the novel coronavirus, and we know what we need to do to stop the spread of virus variants. With global collaboration and rapid data sharing, human society would ultimately win the war against COVID-19. As the SARS-CoV-2 continues evolving and becoming more transmissible between humans and given that it raises serious health concerns worldwide, it is a matter of intense research nowadays. We still do not know whether different SARS-CoV-2 strains, resulting from genetic mutations or adaptive evolution, have affected the transmissibility, susceptibility, and severity of COVID-19 infection. Thus, a convincing knowledge of the SARS-CoV-2 characteristics, including pathogenic mechanism, viral mutation and genetic recombination, its interaction with the host immunopathological response, evolution and genetic diversity, transmission network, and any specific and effective treatment option, may provide a clearer biological understanding of how the virus infects some patients severely, while the majority of patients are only mildly symptomatic or even asymptomatic. This comprehension can also boost a scientist’s ability to successfully design an effective vaccine and take some efficient prophylactic and therapeutic measures, not only for 2019-nCoV but also for future outbreaks of similar coronaviruses.

## Abbreviations

ARDS, acute respiratory distress syndrome; APCs, antigen-presenting cells; CFR, case fatality rate; CRRT, renal replacement therapy; DCs, dendritic cells; CoVs, coronaviruses; ER, estrogens receptors; hACE2, human angiotensin-converting enzyme 2; HLA, human leukocyte antigen; ICTV, International Committee on Taxonomy of Viruses; IFNs, interferons; LUS, lung ultrasound; LncRNAs, long non-coding RNAs; MERS-CoV, Middle East respiratory syndrome coronavirus; RBD, receptor-binding domain; SARS-CoV, severe acute respiratory syndrome coronavirus; SNPs, single nucleotide polymorphisms; WHO, World Health Organization.

## References

[B1] Abdulamir A. S. Hafidh R. R. (2020). The Possible Immunological Pathways for the Variable Immunopathogenesis of COVID-19 Infections Among Healthy Adults, Elderly and Children. Electro. J. Gen. Med. 4 (17), em202. 10.29333/ejgm/7850

[B2] Adams E. R. Anand R. Andersson M. I. Auckland K. Baillie J. K. Barnes E. (2020). Evaluation of Antibody Testing for SARS-Cov-2 Using ELISA and Lateral Flow Immunoassays. MedRxiv. ID: ppmedrxiv-20066407. 10.1101/2020.04.15.20066407

[B3] Ai T. Yang Z. Hou H. Zhan C. Chen C. Lv W. (2020). Correlation of Chest CT and RT-PCR Testing for Coronavirus Disease 2019 (COVID-19) in China: A Report of 1014 Cases. Radiology 296 (2), E32–E40. 10.1148/radiol.2020200642 32101510 PMC7233399

[B4] Akkiz H. (2021). Implications of the Novel Mutations in the SARS-CoV-2 Genome for Transmission, Disease Severity, and the Vaccine Development. Front. Med. (Lausanne) 8, 636532. 10.3389/fmed.2021.636532 34026780 PMC8137987

[B5] Andersen K. G. Rambaut A. Lipkin W. I. Holmes E. C. Garry R. F. (2020). The Proximal Origin of SARS-CoV-2. Nat. Med. 26 (4), 450–452. 10.1038/s41591-020-0820-9 32284615 PMC7095063

[B6] Andreata‐Santos R. Janini L. M. R. Durães‐Carvalho R. (2022). From Alpha to Omicron SARS‐CoV‐2 Variants: what Their Evolutionary Signatures Can Tell Us? J. Med. Virol. 94 (5), 1773–1776. 10.1002/jmv.27555 34978091 PMC9015558

[B7] Anwar M. A. Basith S. Choi S. (2013). Negative Regulatory Approaches to the Attenuation of Toll-like Receptor Signaling. Exp. Mol. Med. 45 (2), e11. 10.1038/emm.2013.28 23429360 PMC3584666

[B8] Asselta R. Paraboschi E. M. Mantovani A. Duga S. (2020). ACE2 and TMPRSS2 Variants and Expression as Candidates to Sex and Country Differences in COVID-19 Severity in Italy. Aging 12 (11), 10087–10098. 10.18632/aging.103415 32501810 PMC7346072

[B9] Atkeson A. (2020). What Will Be the Economic Impact of Covid-19 in the US? Rough Estimates of Disease Scenarios. Cambridge, MA: National Bureau of Economic Research. http://www.nber.org/papers/w26867. 10.3386/w26867

[B10] Ayittey F. K. Ayittey M. K. Chiwero N. B. Kamasah J. S. Dzuvor C. (2020). Economic Impacts of Wuhan 2019‐nCoV on China and the World. J. Med. Virol. 92 (5), 473–475. 10.1002/jmv.25706 32048740 PMC7166799

[B11] Azadbakht J. Khoramian D. Lajevardi Z. S. Elikaii F. Aflatoonian A. H. Farhood B. (2021). A Review on Chest CT Scanning Parameters Implemented in COVID-19 Patients: Bringing Low-Dose CT Protocols into Play. Egypt. J. Radiol. Nucl. Med. 52 (1), 1–10. 10.1186/s43055-020-00400-1

[B12] Azhar E. I. El-Kafrawy S. A. Farraj S. A. Hassan A. M. Al-Saeed M. S. Hashem A. M. (2014). Evidence for Camel-To-Human Transmission of MERS Coronavirus. N. Engl. J. Med. 370 (26), 2499–2505. 10.1056/NEJMoa1401505 24896817

[B13] Bai Y. Yao L. Wei T. Tian F. Jin D.-Y. Chen L. (2020). Presumed Asymptomatic Carrier Transmission of COVID-19. JAMA 323 (14), 1406–1407. 10.1001/jama.2020.2565 32083643 PMC7042844

[B14] Bianchi I. Lleo A. Gershwin M. E. Invernizzi P. (2012). The X Chromosome and Immune Associated Genes. J. Autoimmun. 38 (2-3), J187–J192. 10.1016/j.jaut.2011.11.012 22178198

[B15] Bosch B. J. Van der Zee R. De Haan C. A. M. Rottier P. J. M. (2003). The Coronavirus Spike Protein Is a Class I Virus Fusion Protein: Structural and Functional Characterization of the Fusion Core Complex. J. Virol. 77 (16), 8801–8811. 10.1128/jvi.77.16.8801-8811.2003 12885899 PMC167208

[B16] Broughton J. P. Deng X. Yu G. Fasching C. L. Servellita V. Singh J. (2020). CRISPR-Cas12-based Detection of SARS-CoV-2. Nat. Biotechnol. 38 (7), 870–874. 10.1038/s41587-020-0513-4 32300245 PMC9107629

[B18] Cai X. (2020). An Insight of Comparison between COVID-19 (2019-nCoV Disease) and SARS in Pathology and Pathogenesis. OSF Preprints. 10.31219/osf.io/hw34x

[B19] Cameron M. J. Bermejo-Martin J. F. Danesh A. Muller M. P. Kelvin D. J. (2008). Human Immunopathogenesis of Severe Acute Respiratory Syndrome (SARS). Virus. Res. 133 (1), 13–19. 10.1016/j.virusres.2007.02.014 17374415 PMC7114310

[B20] Cao Z. Zhang Q. Lu X. Pfeiffer D. Jia Z. Song H. (2020). Estimating the Effective Reproduction Number of the 2019-nCoV in China. MedRxiv. 10.1101/2020.01.27.20018952

[B21] Case L. K. Toussaint L. Moussawi M. Roberts B. Saligrama N. Brossay L. (2012). Chromosome Y Regulates Survival Following Murine Coxsackievirus B3 Infection. G3 (Bethesda) 2 (1), 115–121. 10.1534/g3.111.001610 22384388 PMC3276194

[B22] Chan J. F.-W. Yuan S. Kok K.-H. To K. K.-W. Chu H. Yang J. (2020). A Familial Cluster of Pneumonia Associated with the 2019 Novel Coronavirus Indicating Person-To-Person Transmission: a Study of a Family Cluster. The Lancet 395 (10223), 514–523. 10.1016/S0140-6736(20)30154-9 PMC7159286 31986261

[B23] Chan K. W. Wong V. T. Tang S. C. W. (2020). COVID-19: An Update on the Epidemiological, Clinical, Preventive and Therapeutic Evidence and Guidelines of Integrative Chinese-Western Medicine for the Management of 2019 Novel Coronavirus Disease. Am. J. Chin. Med. 48 (3), 737–762. 10.1142/S0192415X20500378 32164424

[B24] Chandra P. Awasthi L. P. (2020). “Plant Virus Taxonomy,” in Applied Plant Virology (Cambridge, MA: Elsevier, Academic Press), 421–434. 10.1016/B978-0-12-818654-1.00029-3

[B25] Channappanavar R. Fett C. Mack M. Ten Eyck P. P. Meyerholz D. K. Perlman S. (2017). Sex-based Differences in Susceptibility to Severe Acute Respiratory Syndrome Coronavirus Infection. J.I. 198 (10), 4046–4053. 10.4049/jimmunol.1601896 PMC5450662 28373583

[B26] Chen J. Jiang Q. Xia X. Liu K. Yu Z. Tao W. (2020). Individual Variation of the SARS-CoV-2 Receptor ACE2 Gene Expression and Regulation. Aging cell 19 (7), e13168. 10.1111/acel.13168 PMC7323071 32558150

[B27] Chen J. Dai L. Kendrick S. Post S. R. Qin Z. (2022). “The Anti-COVID-19 Drug Remdesivir Promotes Oncogenic Herpesviruses Reactivation through Regulation of Intracellular Signaling Pathways,” in Antimicrobial Agents and Chemotherapy. aac-02395. 10.1128/aac.02395-21 PMC8923226 35041508

[B29] Chen N. Zhou M. Dong X. Qu J. Gong F. Han Y. (2020). Epidemiological and Clinical Characteristics of 99 Cases of 2019 Novel Coronavirus Pneumonia in Wuhan, China: a Descriptive Study. The Lancet 395 (10223), 507–513. 10.1016/s0140-6736(20)30211-7 PMC7135076 32007143

[B31] Chien J.-Y. Hsueh P.-R. Cheng W.-C. Yu C.-J. Yang P.-C. (2006). Temporal Changes in Cytokine/chemokine Profiles and Pulmonary Involvement in Severe Acute Respiratory Syndrome. Respirology 11 (6), 715–722. 10.1111/j.1440-1843.2006.00942.x 17052299 PMC7192207

[B32] Chung M. Bernheim A. Mei X. Zhang N. Huang M. Zeng X. (2020). CT Imaging Features of 2019 Novel Coronavirus (2019-nCoV). Radiology 295 (1), 202–207. 10.1148/radiol.2020200230 32017661 PMC7194022

[B33] Coleman C. M. Liu Y. V. Mu H. Taylor J. K. Massare M. Flyer D. C. (2014). Purified Coronavirus Spike Protein Nanoparticles Induce Coronavirus Neutralizing Antibodies in Mice. Vaccine 32 (26), 3169–3174. 10.1016/j.vaccine.2014.04.016 24736006 PMC4058772

[B34] Conti P. Younes A. (2020). Coronavirus COV-19/sars-CoV-2 Affects Women Less Than Men: Clinical Response to Viral Infection. J. Biol. Regul. Homeost Agents 34 (2), 339–343. 10.23812/Editorial-Conti-3 32253888

[B35] Copetti R. (2016). Is Lung Ultrasound the Stethoscope of the New Millennium? Definitely Yes. Ama 45 (1), 80–81. 10.5644/ama2006-124.162 27284804

[B36] Corman V. M. Landt O. Kaiser M. Molenkamp R. Meijer A. Chu D. K. (2020). Detection of 2019 Novel Coronavirus (2019-nCoV) by Real-Time RT-PCR. Eurosurveillance 25 (3), 2000045. 10.2807/1560-7917.es.2020.25.3.2000045 PMC6988269 31992387

[B37] Coutard B. Valle C. de Lamballerie X. Canard B. Seidah N. G. Decroly E. (2020). The Spike Glycoprotein of the New Coronavirus 2019-nCoV Contains a Furin-like Cleavage Site Absent in CoV of the Same Clade. Antivir. Res. 176, 104742. 10.1016/j.antiviral.2020.104742 32057769 PMC7114094

[B38] Cui J. Li F. Shi Z.-L. (2019). Origin and Evolution of Pathogenic Coronaviruses. Nat. Rev. Microbiol. 17 (3), 181–192. 10.1038/s41579-018-0118-9 30531947 PMC7097006

[B39] Dai R. McReynolds S. LeRoith T. Heid B. Liang Z. Ahmed S. (2013). Sex Differences in the Expression of Lupus-Associated miRNAs in Splenocytes from Lupus-Prone NZB/WF1 Mice. Biol. Sex Dif 4 (1), 19. 10.1186/2042-6410-4-19 PMC3843556 24175965

[B40] Dandekar A. A. Perlman S. (2005). Immunopathogenesis of Coronavirus Infections: Implications for SARS. Nat. Rev. Immunol. 5 (12), 917–927. 10.1038/nri1732 16322745 PMC7097326

[B41] Daniloski Z. Jordan T. X. Ilmain J. K. Guo X. Bhabha G. tenOever B. R. (2021). The Spike D614G Mutation Increases SARS-CoV-2 Infection of Multiple Human Cell Types. Elife 10, e65365. 10.7554/eLife.65365 33570490 PMC7891930

[B42] Denina M. Scolfaro C. Silvestro E. Pruccoli G. Mignone F. Zoppo M. (2020). Lung Ultrasound in Children with COVID-19. Pediatrics 146 (1), e20201157. 10.1542/peds.2020-1157 32317309

[B43] Dhama K. Patel S. K. Pathak M. Yatoo M. I. Tiwari R. Malik Y. S. (2020). An Update on SARS-CoV-2/covid-19 with Particular Reference to its Clinical Pathology, Pathogenesis, Immunopathology and Mitigation Strategies. Trav. Med. Infect. Dis. 37, 101755. 10.1016/j.tmaid.2020.101755 PMC7260597 32479816

[B44] Ding Y. Zeng L. Li R. Chen Q. Zhou B. Chen Q. (2017). The Chinese Prescription Lianhuaqingwen Capsule Exerts Anti-influenza Activity through the Inhibition of Viral Propagation and Impacts Immune Function. BMC Complement. Altern. Med. 17 (1), 130. 10.1186/s12906-017-1585-7 28235408 PMC5324200

[B46] Du Toit A. (2020). Outbreak of a Novel Coronavirus. Nat. Rev. Microbiol. 18 (3), 123. 10.1038/s41579-020-0332-0 31988490 PMC7073251

[B47] Ettaboina S. K. Nakkala K. Laddha K. S. (2021). A Mini Review on SARS-COVID-19-2 Omicron Variant (B.1.1.529). Scimed. J. 3 (4), 399–406. 10.28991/scimedj-2021-0304-10

[B48] Fan W. Su Z. Bin Y. Yan-Mei C. Wen W. Zhi-Gang S. (2020). Holmes Edward C., Zhang Yong-Zhen. A New Coronavirus Associated with Human Respiratory Disease in China. Nature 579 (7798), 265–269. 10.1038/s41586-020-2008-3 32015508 PMC7094943

[B49] Farrag M. A. Almajhdi F. N. (2016). Human Respiratory Syncytial Virus: Role of Innate Immunity in Clearance and Disease Progression. Viral Immunol. 29 (1), 11–26. 10.1089/vim.2015.0098 26679242

[B50] Fehr A. R. Perlman S. (2015). “Coronaviruses: an Overview of Their Replication and Pathogenesis,” in Coronaviruses (New York, NY: Springer, Humana Press), 1–23. 10.1007/978-1-4939-2438-7_1 PMC4369385 25720466

[B51] Feng X. Y. Tao X. W. Zeng L. K. Wang W. Q. Li G. (2020). Application of Pulmonary Ultrasound in the Diagnosis of COVID-19 Pneumonia in Neonates. Zhonghua Er Ke Za Zhi 58 (5), 347–350. 10.3760/cma.j.cn112140-20200228-00154 32392948

[B52] Fernandes N. (2020). Economic Effects of Coronavirus Outbreak (COVID-19) on the World Economy. *Available at SSRN 3557504* .

[B53] Fischer J. Jung N. Robinson N. Lehmann C. (2015). Sex Differences in Immune Responses to Infectious Diseases. Infection 43 (4), 399–403. 10.1007/s15010-015-0791-9 25956991

[B54] Freije C. A. Myhrvold C. Boehm C. K. Lin A. E. Welch N. L. Carter A. (2019). Programmable Inhibition and Detection of RNA Viruses Using Cas13. Mol. Cel. 76 (5), 826–837. e811. 10.1016/j.molcel.2019.09.013 PMC7422627 31607545

[B55] Gabay C. (2006). Interleukin-6 and Chronic Inflammation. Arthritis Res. Ther. 8 (S2), S3. 10.1186/ar1917 PMC3226076 16899107

[B56] Garanina E. Martynova E. Davidyuk Y. Kabwe E. Ivanov K. Titova A. (2019). Cytokine Storm Combined with Humoral Immune Response Defect in Fatal Hemorrhagic Fever with Renal Syndrome Case, Tatarstan, Russia. Viruses 11 (7), 601. 10.3390/v11070601 PMC6669480 31269734

[B57] Ge X.-Y. Li J.-L. Yang X.-L. Chmura A. A. Zhu G. Epstein J. H. (2013). Isolation and Characterization of a Bat SARS-like Coronavirus that Uses the ACE2 Receptor. Nature 503 (7477), 535–538. 10.1038/nature12711 24172901 PMC5389864

[B58] Gierer S. Bertram S. Kaup F. Wrensch F. Heurich A. Krämer-Kühl A. (2013). The Spike Protein of the Emerging Betacoronavirus EMC Uses a Novel Coronavirus Receptor for Entry, Can Be Activated by TMPRSS2, and Is Targeted by Neutralizing Antibodies. J. Virol. 87 (10), 5502–5511. 10.1128/jvi.00128-13 23468491 PMC3648152

[B59] Gobeil S. M.-C. Janowska K. McDowell S. Mansouri K. Parks R. Manne K. (2021). D614G Mutation Alters SARS-CoV-2 Spike Conformation and Enhances Protease Cleavage at the S1/S2 junction. Cel Rep. 34 (2), 108630. 10.1016/j.celrep.2020.108630 PMC7762703 33417835

[B60] Gorbalenya A. E. Baker S. C. Baric R. S. de Groot R. J. Drosten C. Gulyaeva A. A. (2020). Severe Acute Respiratory Syndrome-Related Coronavirus: The Species and its Viruses - a Statement of the Coronavirus Study Group. bioRxiv. 10.1101/2020.02.07.937862

[B61] Gralinski L. E. Baric R. S. (2015). Molecular Pathology of Emerging Coronavirus Infections. J. Pathol. 235 (2), 185–195. 10.1002/path.4454 25270030 PMC4267971

[B62] Gralinski L. E. Menachery V. D. (2020). Return of the Coronavirus: 2019-nCoV. Viruses 12 (2), 135. 10.3390/v12020135 PMC7077245 31991541

[B63] Guan W.-j. Ni Z.-y. Hu Y. Liang W.-h. Ou C.-q. He J.-x. (2020a). Clinical Characteristics of 2019 Novel Coronavirus Infection in China. MedRxiv. 10.1101/2020.02.06.20020974

[B64] Guan W.-j. Ni Z.-y. Hu Y. Liang W.-h. Ou C.-q. He J.-x. (2020b). Clinical Characteristics of Coronavirus Disease 2019 in China. N. Engl. J. Med. 382 (18), 1708–1720. 10.1056/nejmoa2002032 32109013 PMC7092819

[B65] Guan Y. Zheng B. J. He Y. Q. Liu X. L. Zhuang Z. X. Cheung C. L. (2003). Isolation and Characterization of Viruses Related to the SARS Coronavirus from Animals in Southern China. Science 302 (5643), 276–278. 10.1126/science.1087139 12958366

[B66] Gui M. Song W. Zhou H. Xu J. Chen S. Xiang Y. (2017). Cryo-electron Microscopy Structures of the SARS-CoV Spike Glycoprotein Reveal a Prerequisite Conformational State for Receptor Binding. Cell Res 27 (1), 119–129. 10.1038/cr.2016.152 28008928 PMC5223232

[B67] Guo L. Wei D. Zhang X. Wu Y. Li Q. Zhou M. (2019). Clinical Features Predicting Mortality Risk in Patients with Viral Pneumonia: the MuLBSTA Score. Front. Microbiol. 10, 2752. 10.3389/fmicb.2019.02752 31849894 PMC6901688

[B68] He X. Hong W. Pan X. Lu G. Wei X. (2021). SARS‐CoV‐2 Omicron Variant: Characteristics and Prevention. MedComm 2 (4), 838–845. 10.1002/mco2.110 PMC8693031 34957469

[B69] Hertanto D. M. Wiratama B. S. Sutanto H. Wungu C. D. K. (2021). Immunomodulation as a Potent COVID-19 Pharmacotherapy: Past, Present and Future. Jir Vol. 14, 3419–3428. 10.2147/jir.s322831 PMC8312605 34321903

[B70] Hoffmann M. Kleine-Weber H. Krüger N. Müller M. Drosten C. Pöhlmann S. (2020a). The Novel Coronavirus 2019 (2019-nCoV) Uses the SARS-Coronavirus Receptor ACE2 and the Cellular Protease TMPRSS2 for Entry into Target Cells. BioRxiv. 10.1101/2020.01.31.929042

[B71] Hoffmann M. Kleine-Weber H. Schroeder S. Krüger N. Herrler T. Erichsen S. (2020b). SARS-CoV-2 Cell Entry Depends on ACE2 and TMPRSS2 and Is Blocked by a Clinically Proven Protease Inhibitor. Cell 181 (5), 271–280. 10.1016/j.cell.2020.02.052 32142651 PMC7102627

[B72] Huang C. Wang Y. Li X. Ren L. Zhao J. Hu Y. (2020). Clinical Features of Patients Infected with 2019 Novel Coronavirus in Wuhan, China. The Lancet 395 (10223), 497–506. 10.1016/s0140-6736(20)30183-5 PMC7159299 31986264

[B73] Huang J.-M. Jan S. S. Wei X. Wan Y. Ouyang S. (2020). Evidence of the Recombinant Origin and Ongoing Mutations in Severe Acute Respiratory Syndrome Coronavirus 2 (SARS-CoV-2). BioRxiv. 10.1101/2020.03.16.993816

[B74] Huang P. Liu T. Huang L. Liu H. Lei M. Xu W. (2020). Use of Chest CT in Combination with Negative RT-PCR Assay for the 2019 Novel Coronavirus but High Clinical Suspicion. Radiology 295 (1), 22–23. 10.1148/radiol.2020200330 32049600 PMC7233360

[B75] Huang Y. Wang S. Liu Y. Zhang Y. Zheng C. Zheng Y. (2020). A Preliminary Study on the Ultrasonic Manifestations of Peripulmonary Lesions of Non-critical Novel Coronavirus Pneumonia (COVID-19). *Available at SSRN 3544750* .

[B76] Jaimes J. A. André N. M. Chappie J. S. Millet J. K. Whittaker G. R. (2020). Phylogenetic Analysis and Structural Modeling of SARS-CoV-2 Spike Protein Reveals an Evolutionary Distinct and Proteolytically Sensitive Activation Loop. J. Mol. Biol. 432 (10), 3309–3325. 10.1016/j.jmb.2020.04.009 32320687 PMC7166309

[B77] Jayk Bernal A. Gomes da Silva M. M. Musungaie D. B. Kovalchuk E. Gonzalez A. Delos Reyes V. (2022). Molnupiravir for Oral Treatment of Covid-19 in Nonhospitalized Patients. N. Engl. J. Med. 386 (6), 509–520. 10.1056/nejmoa2116044 34914868 PMC8693688

[B78] Ji S. Bai Q. Wu X. Zhang D.-W. Wang S. Shen J.-L. (2020). Unique Synergistic Antiviral Effects of Shufeng Jiedu Capsule and Oseltamivir in Influenza A Viral-Induced Acute Exacerbation of Chronic Obstructive Pulmonary Disease. Biomed. Pharmacother. 121, 109652. 10.1016/j.biopha.2019.109652 31734578

[B79] Ji W. Wang W. Zhao X. Zai J. Li X. (2020). Cross‐species Transmission of the Newly Identified Coronavirus 2019‐nCoV. J. Med. Virol. 92 (4), 433–440. 10.1002/jmv.25682 31967321 PMC7138088

[B80] Jiang F. Deng L. Zhang L. Cai Y. Cheung C. W. Xia Z. (2020). Review of the Clinical Characteristics of Coronavirus Disease 2019 (COVID-19). J. Gen. Intern. Med. 35, 1545–1549. 10.1007/s11606-020-05762-w 32133578 PMC7088708

[B81] Jin J.-M. Bai P. He W. Wu F. Liu X.-F. Han D.-M. (2020). Gender Differences in Patients with COVID-19: Focus on Severity and Mortality. Front. Public Health 8, 152. 10.3389/fpubh.2020.00152 32411652 PMC7201103

[B83] Jones S. A. Jenkins B. J. (2018). Recent Insights into Targeting the IL-6 Cytokine Family in Inflammatory Diseases and Cancer. Nat. Rev. Immunol. 18 (12), 773–789. 10.1038/s41577-018-0066-7 30254251

[B84] Kalafat E. Yaprak E. Cinar G. Varli B. Ozisik S. Uzun C. (2020). Lung Ultrasound and Computed Tomographic Findings in Pregnant Woman with COVID‐19. Ultrasound Obstet. Gynecol. 55 (6), 835–837. 10.1002/uog.22034 32249471

[B85] Karlberg J. Chong D. S. Y. Lai W. Y. Y. (2004). Do men Have a Higher Case Fatality Rate of Severe Acute Respiratory Syndrome Than Women Do? Am. J. Epidemiol. 159 (3), 229–231. 10.1093/aje/kwh056 14742282 PMC7110237

[B86] Kawase M. Shirato K. van der Hoek L. Taguchi F. Matsuyama S. (2012). Simultaneous Treatment of Human Bronchial Epithelial Cells with Serine and Cysteine Protease Inhibitors Prevents Severe Acute Respiratory Syndrome Coronavirus Entry. J. Virol. 86 (12), 6537–6545. 10.1128/jvi.00094-12 22496216 PMC3393535

[B87] Kenway-Lynch C. S. Das A. Lackner A. A. Pahar B. (2014). Cytokine/Chemokine Responses in Activated CD4 + and CD8 + T Cells Isolated from Peripheral Blood, Bone Marrow, and Axillary Lymph Nodes during Acute Simian Immunodeficiency Virus Infection. J. Virol. 88 (16), 9442–9457. 10.1128/jvi.00774-14 24920807 PMC4136258

[B88] Kikkert M. (2020). Innate Immune Evasion by Human Respiratory RNA Viruses. J. Innate Immun. 12 (1), 4–20. 10.1159/000503030 31610541 PMC6959104

[B89] Kirchdoerfer R. N. Cottrell C. A. Wang N. Pallesen J. Yassine H. M. Turner H. L. (2016). Pre-fusion Structure of a Human Coronavirus Spike Protein. Nature 531 (7592), 118–121. 10.1038/nature17200 26935699 PMC4860016

[B91] Konermann S. Lotfy P. Brideau N. J. Oki J. Shokhirev M. N. Hsu P. D. (2018). Transcriptome Engineering with RNA-Targeting Type VI-D CRISPR Effectors. Cell 173 (3), 665–676. e614. 10.1016/j.cell.2018.02.033 29551272 PMC5910255

[B92] Kuba K. Imai Y. Rao S. Gao H. Guo F. Guan B. (2005). A Crucial Role of Angiotensin Converting Enzyme 2 (ACE2) in SARS Coronavirus-Induced Lung Injury. Nat. Med. 11 (8), 875–879. 10.1038/nm1267 16007097 PMC7095783

[B93] Kumar S. Thambiraja T. S. Karuppanan K. Subramaniam G. (2021). Omicron and Delta Variant of SARS‐CoV‐2: a Comparative Computational Study of Spike Protein. J. Med. Virol. 94 (4), 1641–1649. 10.1002/jmv.27526 34914115

[B94] Lam T. T.-Y. Jia N. Zhang Y.-W. Shum M. H.-H. Jiang J.-F. Zhu H.-C. (2020). Identifying SARS-CoV-2-Related Coronaviruses in Malayan Pangolins. Nature 583, 282–285. 10.1038/s41586-020-2169-0 32218527

[B95] Lau S. K. P. Woo P. C. Y. Li K. S. M. Huang Y. Tsoi H.-W. Wong B. H. L. (2005). Severe Acute Respiratory Syndrome Coronavirus-like Virus in Chinese Horseshoe Bats. Proc. Natl. Acad. Sci. U.S.A. 102 (39), 14040–14045. 10.1073/pnas.0506735102 16169905 PMC1236580

[B97] Lessler J. Reich N. G. Brookmeyer R. Perl T. M. Nelson K. E. Cummings D. A. (2009). Incubation Periods of Acute Respiratory Viral Infections: a Systematic Review. Lancet Infect. Dis. 9 (5), 291–300. 10.1016/s1473-3099(09)70069-6 19393959 PMC4327893

[B98] Letko M. Marzi A. Munster V. (2020). Functional Assessment of Cell Entry and Receptor Usage for SARS-CoV-2 and Other Lineage B Betacoronaviruses. Nat. Microbiol. 5 (4), 562–569. 10.1038/s41564-020-0688-y 32094589 PMC7095430

[B99] Li C. Yang Y. Ren L. (2020). Genetic Evolution Analysis of 2019 Novel Coronavirus and Coronavirus from Other Species. Infect. Genet. Evol. 82, 104285. 10.1016/j.meegid.2020.104285 32169673 PMC7270525

[B100] Li F. Li W. Farzan M. Harrison S. C. (2005). Structure of SARS Coronavirus Spike Receptor-Binding Domain Complexed with Receptor. Science 309 (5742), 1864–1868. 10.1126/science.1116480 16166518

[B101] Li F. (2008). Structural Analysis of Major Species Barriers between Humans and palm Civets for Severe Acute Respiratory Syndrome Coronavirus Infections. J. Virol. 82 (14), 6984–6991. 10.1128/jvi.00442-08 18448527 PMC2446986

[B102] Li G. De Clercq E. (2020). Therapeutic Options for the 2019 Novel Coronavirus (2019-nCoV). Nat. Rev. Drug Discov. 19, 149–150. 10.1038/d41573-020-00016-0 32127666

[B103] Li Q. Guan X. Wu P. Wang X. Zhou L. Tong Y. (2020). Early Transmission Dynamics in Wuhan, China, of Novel Coronavirus-Infected Pneumonia. N. Engl. J. Med. 382, 1199–1207. 10.1056/NEJMoa2001316 31995857 PMC7121484

[B104] Li W. Shi Z. Yu M. Ren W. Smith C. Epstein J. H. (2005a). Bats Are Natural Reservoirs of SARS-like Coronaviruses. Science 310 (5748), 676–679. 10.1126/science.1118391 16195424

[B105] Li W. Zhang C. Sui J. Kuhn J. H. Moore M. J. Luo S. (2005b). Receptor and Viral Determinants of SARS-Coronavirus Adaptation to Human ACE2. Embo J. 24 (8), 1634–1643. 10.1038/sj.emboj.7600640 15791205 PMC1142572

[B106] Li X. Geng M. Peng Y. Meng L. Lu S. (2020a). Molecular Immune Pathogenesis and Diagnosis of COVID-19. J. Pharm. Anal. 10 (2), 102–108. 10.1016/j.jpha.2020.03.001 32282863 PMC7104082

[B107] Li X. Wang W. Zhao X. Zai J. Zhao Q. Li Y. (2020b). Transmission Dynamics and Evolutionary History of 2019‐nCoV. J. Med. Virol. 92 (5), 501–511. 10.1002/jmv.25701 32027035 PMC7166881

[B108] Li X. Zai J. Zhao Q. Nie Q. Li Y. Foley B. T. (2020c). Evolutionary History, Potential Intermediate Animal Host, and Cross‐species Analyses of SARS‐CoV‐2. J. Med. Virol. 92 (6), 602–611. 10.1002/jmv.25731 32104911 PMC7228310

[B109] Li Y. Zhang J. Wang N. Li H. Shi Y. Guo G. (2020). Therapeutic Drugs Targeting 2019-nCoV Main Protease by High-Throughput Screening. BioRxiv. 10.1101/2020.01.28.922922

[B110] Libert C. Dejager L. Pinheiro I. (2010). The X Chromosome in Immune Functions: when a Chromosome Makes the Difference. Nat. Rev. Immunol. 10 (8), 594–604. 10.1038/nri2815 20651746

[B111] Lichtenstein D. A. Malbrain M. L. N. G. (2017). Lung Ultrasound in the Critically Ill (LUCI): a Translational Discipline. Anaesthesiol Intensive Ther. 49 (5), 430–436. 10.5603/AIT.a2017.0063 29151003

[B112] Lichtenstein D. A. Mezière G. A. (2008). Relevance of Lung Ultrasound in the Diagnosis of Acute Respiratory Failure*: The BLUE Protocol. Chest 134 (1), 117–125. 10.1378/chest.07-2800 18403664 PMC3734893

[B113] Lippi G. Mattiuzzi C. Henry B. M. (2022). Updated Picture of SARS-CoV-2 Variants and Mutations. Diagnosis 9 (1), 11–17. 10.1515/dx-2021-0149 34958184

[B114] Liu G. Lu Y. Thulasi Raman S. N. Xu F. Wu Q. Li Z. (2018). Nuclear-resident RIG-I Senses Viral Replication Inducing Antiviral Immunity. Nat. Commun. 9 (1), 3199. 10.1038/s41467-018-05745-w 30097581 PMC6086882

[B115] Liu J. Ji H. Zheng W. Wu X. Zhu J. J. Arnold A. P. (2010). Sex Differences in Renal Angiotensin Converting Enzyme 2 (ACE2) Activity Are 17β-oestradiol-dependent and Sex Chromosome-independent. Biol. Sex. Differ. 1 (1), 6. 10.1186/2042-6410-1-6 21208466 PMC3010099

[B116] Liu K. Fang Y.-Y. Deng Y. Liu W. Wang M.-F. Ma J.-P. (2020). Clinical Characteristics of Novel Coronavirus Cases in Tertiary Hospitals in Hubei Province. Chin. Med. J. (Engl) 133 (9), 1025–1031. 10.1097/CM9.0000000000000744 32044814 PMC7147277

[B117] Liu L. Iketani S. Guo Y. Chan J. F. Wang M. Liu L. (2021). Striking Antibody Evasion Manifested by the Omicron Variant of SARS-CoV-2. Nature 602 (7898), 676–681. 10.1038/d41586-021-03826-3 35016198

[B118] Liu X. Wang X.-J. (2020). Potential Inhibitors against 2019-nCoV Coronavirus M Protease from Clinically Approved Medicines. J. Genet. Genomics 47 (2), 119–121. 10.1016/j.jgg.2020.02.001 32173287 PMC7128649

[B119] Liu Z. VanBlargan L. A. Bloyet L.-M. Rothlauf P. W. Chen R. E. Stumpf S. (2021). Identification of SARS-CoV-2 Spike Mutations that Attenuate Monoclonal and Serum Antibody Neutralization. Cell host & microbe 29 (3), 477–488. 10.1016/j.chom.2021.01.014 33535027 PMC7839837

[B120] Liu Z. Xiao X. Wei X. Li J. Yang J. Tan H. (2020). Composition and Divergence of Coronavirus Spike Proteins and Host ACE2 Receptors Predict Potential Intermediate Hosts of SARS‐CoV‐2. J. Med. Virol. 92 (6), 595–601. 10.1002/jmv.25726 32100877 PMC7228221

[B122] Lu C.-w. Liu X.-f. Jia Z.-f. (2020). 2019-nCoV Transmission through the Ocular Surface Must Not Be Ignored. The Lancet 395 (10224), e39. 10.1016/s0140-6736(20)30313-5 PMC7133551 32035510

[B123] Lu G. Hu Y. Wang Q. Qi J. Gao F. Li Y. (2013). Molecular Basis of Binding between Novel Human Coronavirus MERS-CoV and its Receptor CD26. Nature 500 (7461), 227–231. 10.1038/nature12328 23831647 PMC7095341

[B124] Lu H. (2020). Drug Treatment Options for the 2019-new Coronavirus (2019-nCoV). Bst 14 (1), 69–71. 10.5582/bst.2020.01020 31996494

[B125] Lu H. Stratton C. W. Tang Y. W. (2020). Outbreak of Pneumonia of Unknown Etiology in Wuhan, China: The Mystery and the Miracle. J. Med. Virol. 92 (4), 401–402. 10.1002/jmv.25678 31950516 PMC7166628

[B126] Lu L. Liu Q. Du L. Jiang S. (2013). Middle East Respiratory Syndrome Coronavirus (MERS-CoV): Challenges in Identifying its Source and Controlling its Spread. Microbes Infect. 15 (8-9), 625–629. 10.1016/j.micinf.2013.06.003 23791956 PMC7110483

[B127] Lu R. Zhao X. Li J. Niu P. Yang B. Wu H. (2020). Genomic Characterisation and Epidemiology of 2019 Novel Coronavirus: Implications for Virus Origins and Receptor Binding. The Lancet 395 (10224), 565–574. 10.1016/s0140-6736(20)30251-8 PMC7159086 32007145

[B128] Lucia C. Federico P.-B. Alejandra G. C. (2020). An Ultrasensitive, Rapid, and Portable Coronavirus SARS-CoV-2 Sequence Detection Method Based on CRISPR-Cas12. bioRxiv. 10.1101/2020.02.29.971127

[B129] Lukassen S. Chua R. L. Trefzer T. Kahn N. C. Schneider M. A. Muley T. (2020). SARS-CoV-2 Receptor ACE2 and TMPRSS2 Are Primarily Expressed in Bronchial Transient Secretory Cells. EMBO J. 39 (10), e105114. 10.15252/embj.20105114 32246845 PMC7232010

[B131] Malkin C. J. Pugh P. J. Jones R. D. Kapoor D. Channer K. S. Jones T. H. (2004). The Effect of Testosterone Replacement on Endogenous Inflammatory Cytokines and Lipid Profiles in Hypogonadal Men. J. Clin. Endocrinol. Metab. 89 (7), 3313–3318. 10.1210/jc.2003-031069 15240608

[B132] Markle J. G. Fish E. N. (2014). SeXX Matters in Immunity. Trends Immunology 35 (3), 97–104. 10.1016/j.it.2013.10.006 24239225

[B133] Martin T. R. Frevert C. W. (2005). Innate Immunity in the Lungs. Proc. Am. Thorac. Soc. 2 (5), 403–411. 10.1513/pats.200508-090js 16322590 PMC2713330

[B134] Maruggi G. Zhang C. Li J. Ulmer J. B. Yu D. (2019). mRNA as a Transformative Technology for Vaccine Development to Control Infectious Diseases. Mol. Ther. 27 (4), 757–772. 10.1016/j.ymthe.2019.01.020 30803823 PMC6453507

[B135] Mayo P. H. Copetti R. Feller-Kopman D. Mathis G. Maury E. Mongodi S. (2019). Thoracic Ultrasonography: a Narrative Review. Intensive Care Med. 45 (9), 1200–1211. 10.1007/s00134-019-05725-8 31418060

[B136] McKibbin W. Fernando R. (2021). The Global Macroeconomic Impacts of COVID-19: Seven Scenarios. Asian Econ. Pap. 20 (2), 1–30. 10.1162/asep_a_00796

[B137] Mescher M. F. Curtsinger J. M. Agarwal P. Casey K. A. Gerner M. Hammerbeck C. D. (2006). Signals Required for Programming Effector and Memory Development by CD8 + T Cells. Immunological Rev. 211 (1), 81–92. 10.1111/j.0105-2896.2006.00382.x 16824119

[B139] Millet J. K. Whittaker G. R. (2014). Host Cell Entry of Middle East Respiratory Syndrome Coronavirus after Two-step, Furin-Mediated Activation of the Spike Protein. Proc. Natl. Acad. Sci. U.S.A. 111 (42), 15214–15219. 10.1073/pnas.1407087111 25288733 PMC4210292

[B140] Morgenstern B. Michaelis M. Baer P. C. Doerr H. W. Cinatl J. Jr (2005). Ribavirin and Interferon-β Synergistically Inhibit SARS-Associated Coronavirus Replication in Animal and Human Cell Lines. Biochem. biophysical Res. Commun. 326 (4), 905–908. 10.1016/j.bbrc.2004.11.128 PMC7092851 15607755

[B141] Mustafa M. I. Makhawi A. M. (2021). SHERLOCK and DETECTR: CRISPR-Cas Systems as Potential Rapid Diagnostic Tools for Emerging Infectious Diseases. J. Clin. Microbiol. 59 (3), e00745–20. 10.1128/JCM.00745-20 33148705 PMC8106734

[B142] Muus C. Luecken M. D. Eraslan G. Waghray A. Heimberg G. Sikkema L. (2020). Integrated Analyses of Single-Cell Atlases Reveal Age, Gender, and Smoking Status Associations with Cell Type-specific Expression of Mediators of SARS-CoV-2 Viral Entry and Highlights Inflammatory Programs in Putative Target Cells. BioRxiv. 10.1101/2020.04.19.049254

[B144] Nelson G. Buzko O. Spilman P. Niazi K. Rabizadeh S. Soon-Shiong P. (2021). Molecular Dynamic Simulation Reveals E484K Mutation Enhances Spike RBD-ACE2 Affinity and the Combination of E484K, K417N and N501Y Mutations (501Y. V2 Variant) Induces Conformational Change Greater Than N501Y Mutant Alone, Potentially Resulting in an Escape Mutant. BioRxiv. 10.1101/2021.01.13.426558

[B145] Nguyen T. M. Zhang Y. Pandolfi P. P. (2020). Virus against Virus: a Potential Treatment for 2019-nCov (SARS-CoV-2) and Other RNA Viruses. Cel Res 30 (3), 189–190. 10.1038/s41422-020-0290-0 PMC7054296 32071427

[B147] Ou X. Zheng W. Shan Y. Mu Z. Dominguez S. R. Holmes K. V. (2016). Identification of the Fusion Peptide-Containing Region in Betacoronavirus Spike Glycoproteins. J. virologyJ Virol 9090 (1212), 55865586–56005600. 10.1128/JVI.00015-16 PMC4886789 27030273

[B148] Pagano A. Numis F. G. Visone G. Pirozzi C. Masarone M. Olibet M. (2015). Lung Ultrasound for Diagnosis of Pneumonia in Emergency Department. Intern. Emerg. Med. 10 (7), 851–854. 10.1007/s11739-015-1297-2 26345533

[B149] Pan Y. Guan H. Zhou S. Wang Y. Li Q. Zhu T. (2020). Initial CT Findings and Temporal Changes in Patients with the Novel Coronavirus Pneumonia (2019-nCoV): a Study of 63 Patients in Wuhan, China. Eur. Radiol. 30 (6), 3306–3309. 10.1007/s00330-020-06731-x 32055945 PMC7087663

[B150] Papanikolaou V. Chrysovergis A. Ragos V. Tsiambas E. Katsinis S. Manoli A. (2022). From Delta to Omicron: S1-Rbd/s2 Mutation/deletion Equilibrium in SARS-CoV-2 Defined Variants. Gene 814, 146134. 10.1016/j.gene.2021.146134 34990799 PMC8725615

[B151] Pardi N. Hogan M. J. Porter F. W. Weissman D. (2018). mRNA Vaccines - a new era in Vaccinology. Nat. Rev. Drug Discov. 17 (4), 261–279. 10.1038/nrd.2017.243 29326426 PMC5906799

[B152] Peiris J. S. M. Guan Y. Yuen K. Y. (2004). Severe Acute Respiratory Syndrome. Nat. Med. 10 (12 Suppl. l), S88–S97. 10.1038/nm1143 15577937 PMC7096017

[B153] Peiris J. S. M. Yuen K. Y. Osterhaus A. D. M. E. Stöhr K. (2003). The Severe Acute Respiratory Syndrome. N. Engl. J. Med. 349 (25), 2431–2441. 10.1056/nejmra032498 14681510

[B154] Phan L. T. Nguyen T. V. Luong Q. C. Nguyen T. V. Nguyen H. T. Le H. Q. (2020). Importation and Human-To-Human Transmission of a Novel Coronavirus in Vietnam. N. Engl. J. Med. 382 (9), 872–874. 10.1056/NEJMc2001272 31991079 PMC7121428

[B155] Pinheiro I. Dejager L. Libert C. (2011). X-chromosome-located microRNAs in Immunity: Might They Explain Male/female Differences? Bioessays 33 (11), 791–802. 10.1002/bies.201100047 21953569

[B156] Poggiali E. Dacrema A. Bastoni D. Tinelli V. Demichele E. Mateo Ramos P. (2020). Can Lung US Help Critical Care Clinicians in the Early Diagnosis of Novel Coronavirus (COVID-19) Pneumonia? Radiology 295 (3), E6. 10.1148/radiol.2020200847 32167853 PMC7233381

[B157] Poutanen S. M. Low D. E. Henry B. Finkelstein S. Rose D. Green K. (2003). Identification of Severe Acute Respiratory Syndrome in Canada. N. Engl. J. Med. 348 (20), 1995–2005. 10.1056/nejmoa030634 12671061

[B158] Prompetchara E. Ketloy C. Palaga T. (2020). Immune Responses in COVID-19 and Potential Vaccines: Lessons Learned from SARS and MERS Epidemic. Asian Pac. J. Allergy Immunol. 38 (1), 1–9. 10.12932/AP-200220-0772 32105090

[B159] Pulliam J. R. C. van Schalkwyk C. Govender N. Gottberg A. v. Cohen C. Groome M. J. (2021). Increased Risk of SARS-CoV-2 Reinfection Associated with Emergence of Omicron in South Africa. medRxiv. 10.1101/2021.11.11.21266068 PMC8995029 35289632

[B160] Quiros-Roldan E. Amadasi S. Zanella I. Degli Antoni M. Storti S. Tiecco G. (2021). Monoclonal Antibodies against SARS-CoV-2: Current Scenario and Future Perspectives. Pharmaceuticals 14 (12), 1272. 10.3390/ph14121272 34959672 PMC8707981

[B161] Rahimi A. Mirzazadeh A. Tavakolpour S. (2021). Genetics and Genomics of SARS-CoV-2: A Review of the Literature with the Special Focus on Genetic Diversity and SARS-CoV-2 Genome Detection. Genomics 113 (1), 1221–1232. 10.1016/j.ygeno.2020.09.059 33007398 PMC7525243

[B162] Raj V. S. Mou H. Smits S. L. Dekkers D. H. W. Müller M. A. Dijkman R. (2013). Dipeptidyl Peptidase 4 Is a Functional Receptor for the Emerging Human Coronavirus-EMC. Nature 495 (7440), 251–254. 10.1038/nature12005 23486063 PMC7095326

[B163] Ramaiah A. Arumugaswami V. (2020). Insights into Cross-Species Evolution of Novel Human Coronavirus SARS-CoV-2 and Defining Immune Determinants for Vaccine Development. BioRxiv. 10.1101/2020.01.29.925867

[B164] Ratti M. Lampis A. Ghidini M. Salati M. Mirchev M. B. Valeri N. (2020). MicroRNAs (miRNAs) and Long Non-coding RNAs (lncRNAs) as New Tools for Cancer Therapy: First Steps from Bench to Bedside. Targ Oncol. 15 (3), 261–278. 10.1007/s11523-020-00717-x PMC7283209 32451752

[B165] Richardson P. Griffin I. Tucker C. Smith D. Oechsle O. Phelan A. (2020). Baricitinib as Potential Treatment for 2019-nCoV Acute Respiratory Disease. The Lancet 395 (10223), e30–e31. 10.1016/s0140-6736(20)30304-4 PMC7137985 32032529

[B166] Røsjø H. Varpula M. Varpula M. Hagve T.-A. Karlsson S. Ruokonen E. (2011). Circulating High Sensitivity Troponin T in Severe Sepsis and Septic Shock: Distribution, Associated Factors, and Relation to Outcome. Intensive Care Med. 37 (1), 77–85. 10.1007/s00134-010-2051-x 20938765 PMC3020309

[B167] Rothe C. Schunk M. Sothmann P. Bretzel G. Froeschl G. Wallrauch C. (2020). Transmission of 2019-nCoV Infection from an Asymptomatic Contact in Germany. N. Engl. J. Med. 382 (10), 970–971. 10.1056/nejmc2001468 32003551 PMC7120970

[B169] Santos C. F. (2020). Reflections about the Impact of the SARS-COV-2/covid-19 Pandemic on Mental Health. Braz. J. Psychiatry 42 (3), 329. 10.1590/1516-4446-2020-0981 32321063 PMC7236153

[B170] Savarino A. Di Trani L. Donatelli I. Cauda R. Cassone A. (2006). New Insights into the Antiviral Effects of Chloroquine. Lancet Infect. Dis. 6 (2), 67–69. 10.1016/s1473-3099(06)70361-9 16439323 PMC7129107

[B171] Schultz M. J. Dunser M. W. Dunser M. W. Dondorp A. M. Adhikari N. K. J. Iyer S. (2017). Current Challenges in the Management of Sepsis in ICUs in Resource-Poor Settings and Suggestions for the Future. Intensive Care Med. 43 (5), 612–624. 10.1007/s00134-017-4750-z 28349179

[B172] Sheahan T. P. Sims A. C. Leist S. R. Schäfer A. Won J. Brown A. J. (2020). Comparative Therapeutic Efficacy of Remdesivir and Combination Lopinavir, Ritonavir, and Interferon Beta against MERS-CoV. Nat. Commun. 11 (1), 222. 10.1038/s41467-019-13940-6 31924756 PMC6954302

[B173] Shen Z. Xiao Y. Kang L. Ma W. Shi L. Zhang L. (2020). Genomic Diversity of Severe Acute Respiratory Syndrome-Coronavirus 2 in Patients with Coronavirus Disease 2019. Clin. Infect. Dis. 71 (15), 713–720. 10.1093/cid/ciaa203 32129843 PMC7108196

[B174] Shimojima M. Fukushi S. Tani H. Taniguchi S. Fukuma A. Saijo M. (2015). Combination Effects of Ribavirin and Interferons on Severe Fever with Thrombocytopenia Syndrome Virus Infection. Virol. J. 12, 181. 10.1186/s12985-015-0412-3 26527529 PMC4630909

[B175] Snijder E. J. Van Der Meer Y. Zevenhoven-Dobbe J. Onderwater J. J. M. Van Der Meulen J. Koerten H. K. (2006). Ultrastructure and Origin of Membrane Vesicles Associated with the Severe Acute Respiratory Syndrome Coronavirus Replication Complex. J. Virol. 80 (12), 5927–5940. 10.1128/JVI.02501-05 16731931 PMC1472606

[B176] Song W. Gui M. Wang X. Xiang Y. (2018). Cryo-EM Structure of the SARS Coronavirus Spike Glycoprotein in Complex with its Host Cell Receptor ACE2. Plos Pathog. 14 (8), e1007236. 10.1371/journal.ppat.1007236 30102747 PMC6107290

[B177] Song Z. Xu Y. Bao L. Zhang L. Yu P. Qu Y. (2019). From SARS to MERS, Thrusting Coronaviruses into the Spotlight. Viruses 11 (1), 59. 10.3390/v11010059 PMC6357155 30646565

[B178] Souyris M. Cenac C. Azar P. Daviaud D. Canivet A. Grunenwald S. (2018). TLR7 Escapes X Chromosome Inactivation in Immune Cells. Sci. Immunol. 3 (19), eaap8855. 10.1126/sciimmunol.aap8855 29374079

[B179] Starr T. N. Greaney A. J. Dingens A. S. Bloom J. D. (2021). Complete Map of SARS-CoV-2 RBD Mutations that Escape the Monoclonal Antibody LY-CoV555 and its Cocktail with LY-CoV016. Cel Rep. Med. 2 (4), 100255. 10.1016/j.xcrm.2021.100255 PMC8020059 33842902

[B180] Sui J. Aird D. R. Tamin A. Murakami A. Yan M. Yammanuru A. (2008). Broadening of Neutralization Activity to Directly Block a Dominant Antibody-Driven SARS-Coronavirus Evolution Pathway. Plos Pathog. 4 (11), e1000197. 10.1371/journal.ppat.1000197 18989460 PMC2572002

[B181] Takashita E. Kinoshita N. Yamayoshi S. Sakai-Tagawa Y. Fujisaki S. Ito M. (2022). Efficacy of Antibodies and Antiviral Drugs against Covid-19 Omicron Variant. New Engl. J. Med. 386 (10), 995–998. 10.1056/nejmc2119407 35081300 PMC8809508

[B182] Tang X. Wu C. Li X. Song Y. Yao X. Wu X. (2020). On the Origin and Continuing Evolution of SARS-CoV-2. Natl. Sci. Rev. 7 (6), 1012–1023. 10.1093/nsr/nwaa036 34676127 PMC7107875

[B183] Taniguchi T. Takaoka A. (2001). A Weak Signal for strong Responses: Interferon-Alpha/beta Revisited. Nat. Rev. Mol. Cel Biol 2 (5), 378–386. 10.1038/35073080 11331912

[B184] Ter Meulen J. Van Den Brink E. N. Poon L. L. M. Marissen W. E. Leung C. S. W. Cox F. (2006). Human Monoclonal Antibody Combination against SARS Coronavirus: Synergy and Coverage of Escape Mutants. Plos Med. 3 (7), e237. 10.1371/journal.pmed.0030237 16796401 PMC1483912

[B185] Tian X. Li C. Huang A. Xia S. Lu S. Shi Z. (2020). Potent Binding of 2019 Novel Coronavirus Spike Protein by a SARS Coronavirus-specific Human Monoclonal Antibody. Emerging microbes & infections 9 (1), 382–385. 10.1080/22221751.2020.1729069 32065055 PMC7048180

[B186] Tiecco G. Storti S. Degli Antoni M. Focà E. Castelli F. Quiros-Roldan E. (2022). Omicron Genetic and Clinical Peculiarities that May Overturn SARS-CoV-2 Pandemic: A Literature Review. Ijms 23 (4), 1987. 10.3390/ijms23041987 35216104 PMC8876558

[B188] Tortorici M. A. Veesler D. (2019). “Structural Insights into Coronavirus Entry,” in Advances in Virus Research (Cambridge, MA: Elsevier, Academic Press), 105, 93–116. 10.1016/bs.aivir.2019.08.002 31522710 PMC7112261

[B189] Tortorici M. A. Walls A. C. Lang Y. Wang C. Li Z. Koerhuis D. (2019). Structural Basis for Human Coronavirus Attachment to Sialic Acid Receptors. Nat. Struct. Mol. Biol. 26 (6), 481–489. 10.1038/s41594-019-0233-y 31160783 PMC6554059

[B190] Totura A. L. Whitmore A. Agnihothram S. Schäfer A. Katze M. G. Heise M. T. (2015). Toll-like Receptor 3 Signaling via TRIF Contributes to a Protective Innate Immune Response to Severe Acute Respiratory Syndrome Coronavirus Infection. mBio 6 (3), e00638–15. 10.1128/mBio.00638-15 26015500 PMC4447251

[B191] Trottein F. Paget C. (2018). Natural Killer T Cells and Mucosal-Associated Invariant T Cells in Lung Infections. Front. Immunol. 9, 1750. eCollection 2018. 10.3389/fimmu.2018.01750 30116242 PMC6082944

[B192] Tsiambas E. Papanikolaou V. Chrysovergis A. Mastronikolis N. Ragos V. Kavantzas N. (2020). Coronavirus in Hematologic Malignancies: Targeting Molecules beyond the Angiotensin-Converting Enzyme 2 (ACE2) wall in COVID-19. Pathol. Oncol. Res. 26 (4), 2823–2825. 10.1007/s12253-020-00810-6 32333199 PMC7182393

[B194] Villaescusa L. Zaragozá F. Gayo-Abeleira I. Zaragozá C. (2022). “A New Approach to the Management of COVID-19. Antagonists of IL-6: Siltuximab,” in Advances in Therapy, 1–23. 10.1007/s12325-022-02042-3 PMC8784859 35072887

[B195] Viveiros A. Rasmuson J. Vu J. Mulvagh S. L. Yip C. Y. Y. Norris C. M. (2021). Sex Differences in COVID-19: Candidate Pathways, Genetics of ACE2, and Sex Hormones. Am. J. Physiology-Heart Circulatory Physiol. 320 (1), H296–H304. 10.1152/ajpheart.00755.2020 PMC8083171 33275517

[B196] Vom Steeg L. G. Klein S. L. (2016). SeXX Matters in Infectious Disease Pathogenesis. Plos Pathog. 12 (2), e1005374. eCollection. 10.1371/journal.ppat.1005374 26891052 PMC4759457

[B197] Walls A. C. Park Y.-J. Tortorici M. A. Wall A. McGuire A. T. Veesler D. (2020). Structure, Function, and Antigenicity of the SARS-CoV-2 Spike Glycoprotein. Cell 181 (2), 281–292. e6. 10.1016/j.cell.2020.02.058 32155444 PMC7102599

[B198] Wan Y. Shang J. Graham R. Baric R. S. Li F. (2020). Receptor Recognition by the Novel Coronavirus from Wuhan: an Analysis Based on Decade-Long Structural Studies of SARS Coronavirus. J. Virol. 94 (7), e00127–20. 10.1128/JVI.00127-20 31996437 PMC7081895

[B200] Wang C. Pan R. Wan X. Tan Y. Xu L. Ho C. S. (2020). Immediate Psychological Responses and Associated Factors during the Initial Stage of the 2019 Coronavirus Disease (COVID-19) Epidemic Among the General Population in China. Int. J. Environ. Res. Public Health 17 (5), 1729. 10.3390/ijerph17051729 PMC7084952 32155789

[B201] Wang M. Cao R. Zhang L. Yang X. Liu J. Xu M. (2020). Remdesivir and Chloroquine Effectively Inhibit the Recently Emerged Novel Coronavirus (2019-nCoV) *In Vitro* . Cel Res 30 (3), 269–271. 10.1038/s41422-020-0282-0 PMC7054408 32020029

[B202] Wang P. Nair M. S. Liu L. Iketani S. Luo Y. Guo Y. (2021). Antibody Resistance of SARS-CoV-2 Variants B.1.351 and B.1.1.7. Nature 593 (7857), 130–135. 10.1038/s41586-021-03398-2 33684923

[B203] Wang W. Tang J. Wei F. (2020). Updated Understanding of the Outbreak of 2019 Novel Coronavirus (2019‐nCoV) in Wuhan, China. J. Med. Virol. 92 (4), 441–447. 10.1002/jmv.25689 31994742 PMC7167192

[B204] Wei X. Xiao Y.-T. Wang J. Chen R. Zhang W. Yang Y. (2020). Sex Differences in Severity and Mortality Among Patients with COVID-19: Evidence from Pooled Literature Analysis and Insights from Integrated Bioinformatic Analysis. arXiv preprint arXiv:2003.13547.

[B205] Williams A. E. Chambers R. C. (2014). The Mercurial Nature of Neutrophils: Still an enigma in ARDS? Am. J. Physiology-Lung Cell Mol. Physiol. 306 (3), L217–L230. 10.1152/ajplung.00311.2013 PMC3920201 24318116

[B206] Winger A. Caspari T. (2021). The Spike of Concern-The Novel Variants of SARS-CoV-2. Viruses 13 (6), 1002. 10.3390/v13061002 34071984 PMC8229995

[B207] Wong C. K. Lam C. W. K. Wu A. K. L. Ip W. K. Lee N. L. S. Chan I. H. S. (2004). Plasma Inflammatory Cytokines and Chemokines in Severe Acute Respiratory Syndrome. Clin. Exp. Immunol. 136 (1), 95–103. 10.1111/j.1365-2249.2004.02415.x 15030519 PMC1808997

[B208] Wong S. K. Li W. Moore M. J. Choe H. Farzan M. (2004). A 193-amino Acid Fragment of the SARS Coronavirus S Protein Efficiently Binds Angiotensin-Converting Enzyme 2. J. Biol. Chem. 279 (5), 3197–3201. 10.1074/jbc.c300520200 14670965 PMC7982343

[B209] World Health Organization (2022b). Available at: https://www.who.int/emergencies/diseases/novel-coronavirus-2019/technical guidance .

[B211] World Health Organization (2022a). WHO Coronavirus (COVID-19) Dashboard. Available at: https://covid19.who.int/?gclid=EAIaIQobChMIm7_G7fWS6gIVlBtBh0jgCAEAAYASAAE gIiavD_BwE .

[B213] Wu C. Chen X. Cai Y. Xia J. a. Zhou X. Xu S. (2020a). Risk Factors Associated with Acute Respiratory Distress Syndrome and Death in Patients with Coronavirus Disease 2019 Pneumonia in Wuhan, China. JAMA Intern. Med. 180 (7), 934–943. 10.1001/jamainternmed.2020.0994 32167524 PMC7070509

[B214] Wu C. Liu Y. Yang Y. Zhang P. Zhong W. Wang Y. (2020b). Analysis of Therapeutic Targets for SARS-CoV-2 and Discovery of Potential Drugs by Computational Methods. Acta Pharmaceutica Sinica B 10 (5), 766–788. 10.1016/j.apsb.2020.02.008 32292689 PMC7102550

[B215] Wu D. Yang X. O. (2020). TH17 Responses in Cytokine Storm of COVID-19: An Emerging Target of JAK2 Inhibitor Fedratinib. J. Microbiol. Immunol. Infect. 53 (3), 368–370. 10.1016/j.jmii.2020.03.005 32205092 PMC7156211

[B216] Wu F. Zhao S. Yu B. Chen Y.-M. Wang W. Song Z.-G. (2020). A New Coronavirus Associated with Human Respiratory Disease in China. Nature 579 (7798), 265–269. 10.1038/s41586-020-2008-3 32015508 PMC7094943

[B217] Wu Z. McGoogan J. M. (2020). Characteristics of and Important Lessons from the Coronavirus Disease 2019 (COVID-19) Outbreak in China. Jama 323 (13), 1239–1242. 10.1001/jama.2020.2648 32091533

[B218] Xie X. Zhong Z. Zhao W. Zheng C. Wang F. Liu J. (2020). Chest CT for Typical Coronavirus Disease 2019 (COVID-19) Pneumonia: Relationship to Negative RT-PCR Testing. Radiology 296, E41–E200343. 10.1148/radiol.2020200343 32049601 PMC7233363

[B219] Xu H. Zhong L. Deng J. Peng J. Dan H. Zeng X. (2020). High Expression of ACE2 Receptor of 2019-nCoV on the Epithelial Cells of Oral Mucosa. Int. J. Oral Sci. 12 (1), 8–5. 10.1038/s41368-020-0074-x 32094336 PMC7039956

[B220] Xu R.-H. He J.-F. Evans M. R. Peng G.-W. Field H. E. Yu D.-W. (2004). Epidemiologic Clues to SARS Origin in China. Emerg. Infect. Dis. 10 (6), 1030–1037. 10.3201/eid1006.030852 15207054 PMC3323155

[B221] Xu Z. Shi L. Wang Y. Zhang J. Huang L. Zhang C. (2020). Pathological Findings of COVID-19 Associated with Acute Respiratory Distress Syndrome. Lancet Respir. Med. 8 (4), 420–422. 10.1016/s2213-2600(20)30076-x 32085846 PMC7164771

[B222] Yang J.-K. Lin S.-S. Ji X.-J. Guo L.-M. (2010). Binding of SARS Coronavirus to its Receptor Damages Islets and Causes Acute Diabetes. Acta Diabetol. 47 (3), 193–199. 10.1007/s00592-009-0109-4 19333547 PMC7088164

[B223] Yang X. Yu Y. Xu J. Shu H. Xia J. a. Liu H. (2020). Clinical Course and Outcomes of Critically Ill Patients with SARS-CoV-2 Pneumonia in Wuhan, China: a Single-Centered, Retrospective, Observational Study. Lancet Respir. Med. 8 (5), 475–481. 10.1016/S2213-2600(20)30079-5 32105632 PMC7102538

[B224] Yang Y. Yang M. Yuan J. Wang F. Wang Z. Li J. (2020). Laboratory Diagnosis and Monitoring the Viral Shedding of SARS-CoV-2 Infection. The Innovation 1 (3), 100061. 10.1016/j.xinn.2020.100061 33169119 PMC7609236

[B225] Yang Y. Zhang L. Geng H. Deng Y. Huang B. Guo Y. (2013). The Structural and Accessory Proteins M, ORF 4a, ORF 4b, and ORF 5 of Middle East Respiratory Syndrome Coronavirus (MERS-CoV) Are Potent Interferon Antagonists. Protein Cell 4 (12), 951–961. 10.1007/s13238-013-3096-8 24318862 PMC4875403

[B226] Yang Z.-y. Kong W.-p. Huang Y. Roberts A. Murphy B. R. Subbarao K. (2004). A DNA Vaccine Induces SARS Coronavirus Neutralization and Protective Immunity in Mice. Nature 428 (6982), 561–564. 10.1038/nature02463 15024391 PMC7095382

[B227] Yao H.-P. Lu X. Chen Q. Xu K. Chen Y. Cheng L. (2020). Patient-derived Mutations Impact Pathogenicity of SARS-CoV-2. medRxiv. *CELL-D-20-01124* . 10.1101/2020.04.14.20060160

[B228] Yao H. Chen J.-H. Xu Y.-F. (2020). Patients with Mental Health Disorders in the COVID-19 Epidemic. The Lancet Psychiatry 7 (4), e21. 10.1016/s2215-0366(20)30090-0 32199510 PMC7269717

[B229] Ye Z. Zhang Y. Wang Y. Huang Z. Song B. (2020). Chest CT Manifestations of New Coronavirus Disease 2019 (COVID-19): a Pictorial Review. Eur. Radiol. 30 (8), 4381–4389. 10.1007/s00330-020-06801-0 32193638 PMC7088323

[B231] Yuan M. Liu H. Wu N. C. Wilson I. A. (2021). Recognition of the SARS-CoV-2 Receptor Binding Domain by Neutralizing Antibodies. Biochem. biophysical Res. Commun. 538, 192–203. 10.1016/j.bbrc.2020.10.012 PMC7547570 33069360

[B232] Yuan Y. Cao D. Zhang Y. Ma J. Qi J. Wang Q. (2017). Cryo-EM Structures of MERS-CoV and SARS-CoV Spike Glycoproteins Reveal the Dynamic Receptor Binding Domains. Nat. Commun. 8 (1), 15092–15099. 10.1038/ncomms15092 28393837 PMC5394239

[B234] Zhang T. Wu Q. Zhang Z. (2020). Probable Pangolin Origin of SARS-CoV-2 Associated with the COVID-19 Outbreak. Curr. Biol. 30 (7), 1346–1351. e2. 10.1016/j.cub.2020.03.022 32197085 PMC7156161

[B235] Zhang Y. Ma Z. F. (2020). Impact of the COVID-19 Pandemic on Mental Health and Quality of Life Among Local Residents in Liaoning Province, China: A Cross-Sectional Study. Ijerph 17 (7), 2381. 10.3390/ijerph17072381 PMC7177660 32244498

[B236] Zheng J. (2020). SARS-CoV-2: an Emerging Coronavirus that Causes a Global Threat. Int. J. Biol. Sci. 16 (10), 1678–1685. 10.7150/ijbs.45053 32226285 PMC7098030

[B237] Zhong N. (2004). Management and Prevention of SARS in China. Phil. Trans. R. Soc. Lond. B 359 (1447), 1115–1116. 10.1098/rstb.2004.1491 15306397 PMC1693398

[B238] Zhou F. Yu T. Du R. Fan G. Liu Y. Liu Z. (2020). Clinical Course and Risk Factors for Mortality of Adult Inpatients with COVID-19 in Wuhan, China: a Retrospective Cohort Study. The Lancet 395 (10229), 1054–1062. 10.1016/S0140-6736(20)30566-3 PMC7270627 32171076

[B239] Zhou J.-h. Wang Y.-n. Chang Q.-y. Ma P. Hu Y. Cao X. (2018). Type III Interferons in Viral Infection and Antiviral Immunity. Cell Physiol Biochem 51 (1), 173–185. 10.1159/000495172 30439714

[B240] Zhou J. Chu H. Li C. Wong B. H.-Y. Cheng Z.-S. Poon V. K.-M. (2014). Active Replication of Middle East Respiratory Syndrome Coronavirus and Aberrant Induction of Inflammatory Cytokines and Chemokines in Human Macrophages: Implications for Pathogenesis. J. Infect. Dis. 209 (9), 1331–1342. 10.1093/infdis/jit504 24065148 PMC7107356

[B241] Zhou P. Yang X.-L. Wang X.-G. Hu B. Zhang L. Zhang W. (2020). A Pneumonia Outbreak Associated with a New Coronavirus of Probable Bat Origin. Nature 579 (7798), 270–273. 10.1038/s41586-020-2012-7 32015507 PMC7095418

[B242] Zhu J. Yamane H. Paul W. E. (2010). Differentiation of Effector CD4 T Cell Populations. Annu. Rev. Immunol.Annu Rev. Immunol. 2828, 445445–448989. 10.1146/annurev-immunol-030409-101212 PMC3502616 20192806

[B243] Zhu N. Zhang D. Wang W. Li X. Yang B. Song J. (2020). A Novel Coronavirus from Patients with Pneumonia in China, 2019. N. Engl. J. Med. 382 (8), 727–733. 10.1056/NEJMoa2001017 31978945 PMC7092803

[B244] Zumla A. Chan J. F. W. Azhar E. I. Hui D. S. C. Yuen K.-Y. (2016). Coronaviruses - Drug Discovery and Therapeutic Options. Nat. Rev. Drug Discov. 15 (5), 327–347. 10.1038/nrd.2015.37 26868298 PMC7097181

